# A Platform for Sustainable Scale: The Challenge Initiative’s Innovative Approach to Scaling Proven Interventions

**DOI:** 10.9745/GHSP-D-22-00167

**Published:** 2024-05-21

**Authors:** Clea Finkle, Kim Martin, Ian Salas, Jessica Mirano, Lisa Mwaikambo, Kojo Lokko, Jose Rimon

**Affiliations:** a Independent consultant, Seattle, WA, USA.; b Bill & Melinda Gates Institute for Population and Reproductive Health, Bloomberg School of Public Health, Johns Hopkins University, Baltimore, MD, USA.; c Johns Hopkins Center for Communication Programs, Baltimore, MD, USA.

## Abstract

**Introduction:**

The global health community continues to face barriers in scaling up evidence-based interventions for widespread adoption. Although many effective interventions have been developed over the years, expanding their reach to benefit broader populations has happened slowly or not at all.

**Overview:**

The Challenge Initiative (TCI) is a nontraditional development platform that supports local urban governments to rapidly scale up proven family planning (FP) and adolescent and youth sexual and reproductive health (AYSRH) interventions for the urban poor. TCI prioritizes sustainability and local ownership and uses a health systems approach when planning for and managing scale. TCI strengthens urban health systems with seed funding, coaching, and technical assistance (TA), and TCI University houses “how-to” guidance and tools for implementing the interventions. In turn, local governments commit political will and financial and human resources while using TCI coaching to integrate interventions into routine practice and systems to achieve widespread and sustained impact at scale.

**Results:**

As of June 2021, TCI has supported 104 local governments across 11 countries in scaling up effective FP and AYSRH interventions, while also mobilizing about US$28 million from those local governments to facilitate their implementation. TCI has increased capacity and bolstered urban health systems, with 39 local governments “graduating” from TCI support and 2.02 million additional FP clients across 4 regional TA hubs.

**Conclusion:**

TCI aims to change how local governments coordinate, finance, and implement proven interventions to improve access to quality FP information and services. With built-in incentives for local governments, partners, and donors to participate, TCI is generating significant learning on how local governments can realize sustainable scale and demonstrating how organizations like TCI that facilitate governments to scale up effective interventions can accelerate the scale-up of these interventions across multiple geographies.

## Abstract (precis)

The Challenge Initiative’s innovative approach to scale prioritizes local government ownership and meaningful leadership of key local stakeholders to implement high-impact interventions with built-in sustainability by strengthening local capacity and health systems.

## Teaser

**Key Findings:**

- There is a great need to scale up proven health interventions for the urban poor, including improving access to contraception, in a sustainable and cost-efficient way.
- To enable the scale-up of intervention packages across multiple geographies, The Challenge Initiative (TCI) platform includes regional technical assistance hubs that meaningfully engage local stakeholders and systems and a global partner that raises funds; supports shared learning; and upholds consistent practices, processes, and standards across all hubs.
- Government self-selection and commitment of funds to participate in TCI are critical to the ownership and sustainability of the change process; they also complement and reinforce local leadership in program design, implementation, and management.

**Key Implications:**

- Leadership, management, and coordination functions are critical areas for capacity building and successful programming, on par with support to strengthen technical capacity.
- Scale measures, including reach, uptake (adoption), and sustainability should be designed for local governments to monitor progress without external assistance.
- Strengthening sustainable systems for scaling effective interventions requires alignment of mission, management, and leadership with programmatic success.

## INTRODUCTION

The global health community continues to face barriers in scaling up evidence-based interventions for widespread adoption. While many effective interventions have been developed over the years, expanding their reach to benefit broader populations has taken place slowly or not at all. Some of the reasons behind failed scaling efforts include underestimating the importance of politics and policy, failing to ensure local government ownership from the beginning, misjudging the time and resources needed for successful scale-up with sustainable impact, and the failure to adapt or make portable an innovation shown to be effective in one context to a new context.^1^^–^^4^

ExpandNet and the World Health Organization define scale-up as “efforts to increase the impact of innovations that have been successfully tested in pilot or experimental projects so as to benefit more people and to foster policy and program development on a lasting basis.”^5^

The last 2 decades have seen a rapidly maturing literature on scale-up theory and practice, motivated in part by the urgency to achieve the Millennium Development Goals and the Sustainable Development Goals. The literature is replete with learnings about scale-up success factors, barriers, and challenges. A growing number of tools, empirical studies, and conceptual frameworks have been developed to guide strategic thinking about the process, conditions, and outcomes of scale-up^2^^,^^4^^–^^12^ spanning a wide array of disciplines, including agriculture; water, sanitation, and hygiene; health; poverty; education; climate change; and youth employment among others. More recently, a consensus appears to be emerging on several critical fronts, including the general stages entailed before and during the scale-up process, the critical role of adaptive management and “learning by doing,” the importance of close and continuous engagement with health system stakeholders, the need to design for sustainability, and others.^2^^,^^4^^,^^5^^,^^9^^–^^11^^,^^13^

In this article, we describe an ambitious family planning (FP) scaling platform, The Challenge Initiative (TCI), which is currently implemented in 11 countries to rapidly scale up proven FP interventions for the urban poor. We explain the principal components of the platform and how its design elements are informed by the scaling field’s learnings as well as some of its outstanding questions. Rather than present TCI as having “resolved” these questions, we share its experience and reflections to contribute to the learnings in the wider field of scale-up theory and practice for FP, adolescent and youth sexual and reproductive health (AYSRH), and other health areas, by addressing the following 5 scaling questions.

1. What approaches build capacity and strengthen government systems to aid scale-up?
2. What incentives facilitate the adoption and sustained commitment to an innovation?
3. How can scaling be accelerated?
4. What measures and data sources support sustainable scale-up?
5. Can donor norms be transformed to finance scale-up?

We share TCI’s experience to contribute to the learnings in the field of scale-up theory and practice for FP and AYSRH.

## THE TCI PLATFORM

### Background

TCI builds on the success of the Urban Reproductive Health Initiative (URHI), a Bill & Melinda Gates Foundation (Gates Foundation)—funded urban FP project implemented in India, Kenya, Nigeria, and Senegal from 2010 to 2015. URHI was a proof of concept that aimed to test a set of supply, demand, and advocacy interventions to improve quality, accessibility, and contraceptive services for the urban poor. The rationale for the focus on low-income urban communities is the demographic importance of this growing population, their poor health outcomes,^14^^,^^15^ the lack of scaled approaches for reaching them with FP services, and the paucity of philanthropic investments to do so. During the first 3 years of URHI, intervention packages were tested in 4 cities in each country, with each country having a different package. With technical assistance (TA) to support a systematic approach to scale from ExpandNet^16^ and findings from the midterm evaluation, streamlined packages of URHI’s interventions were tested for scalability in additional cities in each country, with cities taking a leadership role and contributing some of their own funding.

An external evaluation of URHI found positive associations between the interventions and contraceptive uptake and identified interventions associated with the highest impact on contraceptive uptake.^17^^–^^21^ Equally important, URHI’s pilot and scalability phases generated important learnings that would inform the design of the TCI model, including the feasibility of local governments to lead—and contribute their own resources to—the introduction of a set of innovations while still achieving positive outcomes in contraceptive uptake.^22^^,^^23^

In 2016, the Gates Foundation funded TCI to serve as a platform to support governments to sustainably scale URHI’s proven interventions in urban areas of East Africa, India, Nigeria, and francophone West Africa. Unlike URHI, TCI’s mandate from the outset identified sustainability as a key performance outcome for the platform’s rapid scale-up of proven FP approaches to cities across multiple countries. Originally designed to support the scaling of URHI’s evidence-based interventions, TCI has evolved into a platform for also scaling global High Impact Practices (HIPs)^24^—a set of evidence-based practices representing global expert consensus on what works in FP programming—along with other approaches that have shown promise for increasing access to FP by the urban poor.

### Organization and Management Structure

TCI’s management structure was designed to facilitate rapid scale-up of FP interventions in urban areas across multiple countries. The Bill & Melinda Gates Institute for Population and Reproductive Health based at Johns Hopkins Bloomberg School of Public Health (Gates Institute) oversees regional TA “hubs” that support governments to scale up the high-impact interventions in their regions. Until 2020, the platform included 4 TA hubs, each managed by different implementing partners: Jhpiego in East Africa, Population Services International in India, Johns Hopkins Center for Communication Programs in Nigeria, and IntraHealth International in francophone West Africa. Three of these hub partners led the original URHI project in the same region with significant continuity of staff. In 2020, a fifth TA hub was established in the Philippines, led by the Philippine-based Zuellig Family Foundation, and in 2022, a sixth hub was added in Pakistan, led by Greenstar Social Marketing, also locally based.

In its role as the global platform manager, the Gates Institute ensures standardization, coordination, and quality control across all hubs, while also engaging donors to mobilize funding for the platform to expand to new geographies. The global platform manager has proven valuable in managing the relationships with donors (i.e., reporting and day-to-day management), developing and continually updating TCI University (TCI-U) to reflect and share hub-specific learnings with other hubs and the global community, and ensuring adherence to TCI’s core principles, including that local leaders drive the change process. For example, the global platform manager guided the Nigeria hub to transition demand generation work from a subcontractor to the states’ own government-run social and behavior change communication committees to strengthen sustainability. The global platform manager also supported hubs in streamlining coaching design to enable institutionalization within government structures. For example, the India hub was advised to shift from direct implementation to building the capacity of urban primary health center–based auxiliary nurse midwives to manage accredited social health activists.

The regional TA hubs engage directly with local stakeholders and provide guidance to participating governments to identify and adapt the interventions for the local context and to develop scale-up plans aligned with the resources, policies, and guidance of their health system; manage technical and leadership coaching to build capacity; and lead regional advocacy and knowledge management.

Taken together, the TCI platform performs intermediary functions critical for successful scale-up: strategic planning, fundraising, advocacy and marketing, convening and coordinating of stakeholders, TA, and change management.^25^^,^^26^ In this way, the TCI platform acts as an intermediary organization (the organization supporting a government to scale and integrate an innovation into its systems, policies, and practices) with local governments as hosts (the organization expected to implement the intervention for the long term, typically governments, nongovernmental organizations, commercial markets, or hybrid combinations). Despite its importance for scaling, intermediary support to a government is frequently overlooked or underestimated by funders.^6^

The TCI platform performs intermediary functions critical for successful scale-up: strategic planning, fundraising, advocacy and marketing, convening and coordinating of stakeholders, TA, and change management.

### How TCI Works

TCI’s primary engagement is with city governments, although the initiative also partners with other administrative levels (e.g., county/subcounty in Kenya and districts in Tanzania and Uganda, depending on country context) that have a determining role in the financial, administrative, and service delivery functions of their urban populations. Within an urban area, service delivery support centers on the highest-volume facilities. Under URHI, the implementing partners selected which cities to work in based on size, potential impact, and other criteria. Under TCI, participating governments “self-select” to join, as demand driven is a key guiding principle for TCI (Box 1). They learn about TCI through its marketing efforts, which target subnational governments with large urban populations that are politically committed, able to make a financial contribution, and demonstrate a minimum level of health system capacity (i.e., number of trained providers for all FP methods, status of the supply chain system, supportive supervision structure, and other indicators). Interested governments submit a formal letter of inquiry along with a pledge of funding and political commitment to deliver the FP and/or AYSRH interventions. If approved, they draw from TCI’s “Challenge Fund” for seed funding to supplement their own financial commitments; organize implementation teams; and coordinate with cross-departmental government partners as well as civil society (e.g., youth groups and community gatekeepers) and private sector organizations, as relevant. Throughout this process, governments access TCI-U, which provides tailored in-person and virtual TA coaching (Supplement 1) that tapers off in intensity as local capacity grows and local stakeholders assume full control of implementation; FP and AYSRH toolkits that house the interventions with practical implementation guidance and tools; and a virtual community of practice to enable shared learning. Local governments are also coached to improve the quality of locally generated service statistics data to monitor and improve performance and review and adjust plans each year.

BOX 1 The Challenge Initiative’s Key Principles for Mainstreaming High-Impact Interventions Into Urban Government Programs

- Support local ownership and self-reliance through demand-driven model
- Adapt interventions to local context
- Transfer capacity through technical and managerial coaching and TCI University
- Leverage existing systems to harmonize strategies, plans, funding, and technical coaching
- Use data-driven decision making and adaptive management through continuous learning

The period of local government engagement with TCI is typically 3–5 years—from the initial planning phase to early implementation, then “surge” (when all elements of an intervention package are being implemented), pregraduation (when TCI gradually reduces its support), and graduation and beyond (when TCI continues to monitor performance and provides coaching “on demand”). The intensity of TA support that TCI provides to local governments throughout these phases is designed to ensure greater self-sustained improvements in urban health systems and increased use of modern contraception, with a focus on low-income urban populations.

The intensity of TA support that TCI provides to local governments throughout implementation phases is designed to ensure greater self-sustained improvements in urban health systems and increased use of modern contraception, with a focus on low-income urban populations.

### Intervention Package

TCI supports local governments to adopt and institutionalize a core package of demand generation, service delivery, and advocacy interventions adapted to the local context and housed in global and hub-specific toolkits on TCI-U. These core interventions align with 10 global HIPs, with hub toolkits also including additional interventions applicable to their regional context (Box 2). A city’s choice of interventions is informed by the key areas of need identified through a rapid landscape assessment conducted in the initial planning phase through a participatory process. TCI-U also includes AYSRH interventions to address provider bias, promote youth-friendly cities, engage pharmacies and drug shops, and use other approaches that enable younger women and men to access contraceptives.^27^

BOX 2 Global High Impact Practices That Align Directly With The Challenge Initiative’s Family Planning and Adolescent and Youth Sexual and Reproductive Health Interventions

**Service Delivery**

- Community health workers
- Immediate postpartum family planning
- Mobile outreach services
- Pharmacies and drug shops

**Enabling Environment**

- Domestic public financing
- Galvanizing commitment
- Leaders and managers

**Social and Behavior Change**

- Mass media
- Community group engagement

**High Impact Practice Enhancement**

- Adolescent-responsive contraceptive services

In addition to technical (intervention-focused) coaching on FP and AYSRH interventions, TCI provides coaching assistance to strengthen a local government’s leadership, management, and coordination functions, financial management and monitoring, adaptation of interventions, and data for decision making—what TCI refers to as “essentials” for successful programming. These essentials have become an increasingly prominent focus of coaching support, based on learnings that coaching at higher levels of the health system—such as state, city, and local government area administrative levels rather than only at the facility/provider level—is required to ensure sufficient capacity to scale and potential to sustain systems level capability to maintain quality FP programming.

### Coaching

TCI builds government capacity through targeted coaching on the technical aspects of the intervention package as well as on effective management and health governance to address capacity gaps. TCI coaching is conducted by local and regional master coaches who cascade the evidence-based solutions and guidance to municipal health personnel and managers to strengthen local health systems to implement the selected interventions. Initially, TCI hub staff serve as master coaches but transfer this role to local experts (including from the government) with strong FP and AYSRH experience. In consultation with TCI, city implementation teams identify managers and implementers for training as coaches relevant to specific proven interventions. For service delivery interventions, for example, TCI and local government counterparts review the list of those who currently provide supportive supervision and are considered master trainers of the government as a good starting point for resource persons to be trained as TCI coaches within the public health system. Once coached by TCI, master trainers in turn coach individuals from health system leadership (e.g., political leaders and ministry of health leadership); lower-level managers (e.g., medical officers-in-charge, nurses-in-charge/matrons, FP/RH coordinators, FP officers, urban health officers, health educators, monitoring and evaluation officers, and adolescent health officers); and implementers (e.g., health workers such as FP, health district, and facility-based personnel, and community health workers). As of June 2021, more than 3,300 coaches have been trained across 4 regional TA hubs.

### Monitoring and Learning for Scale

TCI’s key outcome measures are anchored in platform scale, contraceptive uptake, health systems strengthening for sustainability, and platform cost-efficiency. Data is triangulated from several sources to collect these measures: the government’s service statistics as captured in its health management information system (HMIS); project records and available surveys; TCI’s RAISE (Reflection and Action to Improve Self-Reliance and Effectiveness) maturity model tool, which uses a participatory approach to self-assess sustainability progress in real time^28^; the Most Significant Change (MSC) technique,^29^ which supports adaptive management, routine monitoring, and cross-learning efforts locally and globally; and regular “pause-and-reflect” sessions that generate additional insights and evidence on the scaling pathway. In a large-scale program such as TCI, high-frequency program monitoring is critical to determine where further support is needed and where lessons can be learned, shared, and successfully replicated.

TCI’s key outcome measures are anchored in platform scale, contraceptive uptake, health systems strengthening for sustainability, and platform cost-efficiency.

Regularly scheduled monthly data review meetings, quarterly MSC selection meetings, and periodic pause-and-reflect exercises form the backbone of TCI’s adaptive management approach, which aims to continuously track changes, facilitate learning and responsive feedback, and capture program contributions to intended and unintended outcomes.

### Platform Scale

**FIGURE 1:**
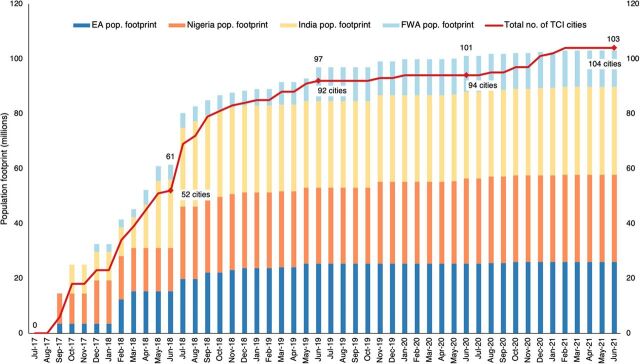
The Challenge Initiative Scaling Over Time, 2017–2021, by Population and Number of Cities^a^ Abbreviations: EA, East Africa; FWA, francophone West Africa; TCI, The Challenge Initiative. ^a^ The cumulative number of cities is indicated by the line. Population footprint by hub.

TCI monitors 2 main platform-level performance measures monthly: population footprint (the aggregate population of TCI-supported cities) and number of cities engaged. These indicators represent the reach of the platform and how it is distributed across the regional hubs over time. Figure 1 shows the rapid scale that took place over 5 years, with expansion slowing substantially beginning in 2018 to secure additional funding, assess TCI’s progress to date, and then, in response to COVID-19.

As of June 2021, TCI has partnered with 104 local governments in 11 countries (Benin, Burkina Faso, Cote d’Ivoire, India, Kenya, Niger, Nigeria, the Philippines, Senegal, Tanzania, and Uganda) to implement FP and AYSRH interventions for low-income urban communities.

### Contraceptive Uptake

TCI has 3 main indicators for tracking changes in contraceptive uptake: client volume, derived number of additional clients, and net contraceptive uptake. These indicators were developed by reviewing existing data sources—namely, the country’s own HMIS and population census data—and adapting them to meet the needs of local governments to measure and monitor the impact of scaling proven FP interventions.^30^ TCI’s coaching to strengthen data systems and use helps local government staff provide timely, accurate, and complete reporting of data to HMIS; make more effective use of their own HMIS data for program monitoring and internal decision making; and advocate for continued and increased funding for FP interventions based on data.

A continuing challenge, particularly in francophone West Africa, is weak data systems that jeopardize sustained government use of HMIS data, a challenge noted by an external TCI program review conducted in 2020 (described in more detail in the External Program Review section). Another challenge is disaggregating data to segment youth and the urban poor, identifiers typically not captured in routine data systems.

As of June 2021, overall data showed an increasing trend in FP clients in TCI cities, especially for long-acting reversible contraceptives. An effective number of 2.65 million clients received a modern FP method across 104 TCI cities for the 12-month period ending in June 2021, 44% higher than the baseline period (i.e., the 12-month period before partnering with TCI). Across all cities, this translated to an estimated (derived) number of 2.02 million additional clients from the launch of a city’s partnership with TCI through June 2021 and is equivalent to 2.49 additional clients per 100 women of reproductive age per year (net contraceptive uptake).

**FIGURE 2:**
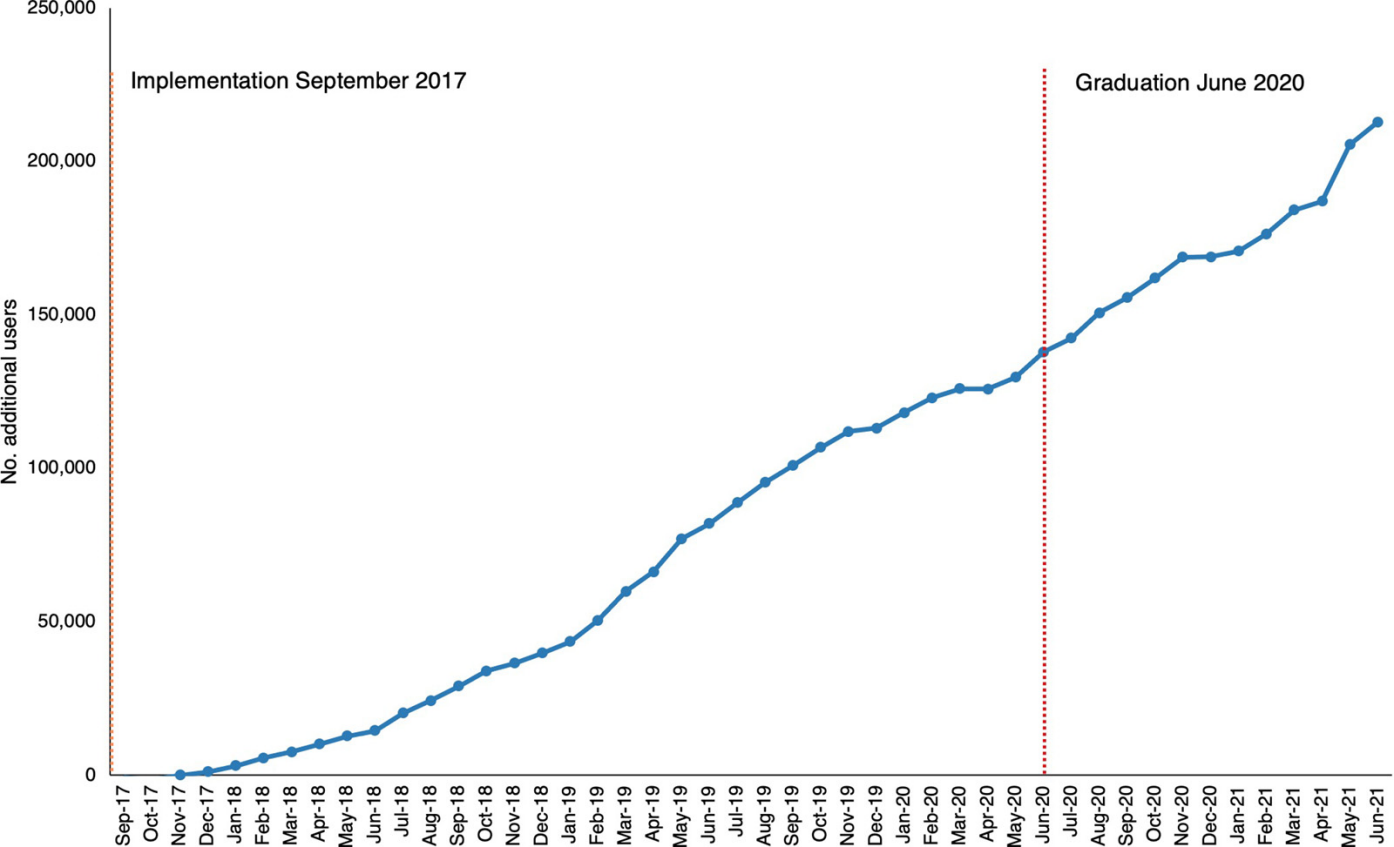
Pre- and Postgraduation Trends in Additional Family Planning Clients in 5 East African Cities

TCI has been closely monitoring contraceptive uptake in graduated cities. As of June 2021 (the end date for data in this article), there were 5 graduated cities, all in East Africa, with at least 1 year of data on postgraduation contraceptive uptake. Figure 2 shows a continuing upward trend in additional FP clients from the start of these cities’ engagement with TCI (September 2017) through graduation (June 2020) to 1 year postgraduation. Supplement 2 provides a preliminary analysis of additional FP client trends postgraduation for more cities through February 2022.

### Sustainability

TCI identified several predictors of sustainability that helped inform its approach to measuring local government progress toward graduation and greater self-reliance. From these predictors, it developed RAISE, a maturity model tool used by local governments to self-assess progress toward sustainability for implementing the intervention package.^31^ Each quarter, a city’s key health personnel use RAISE to monitor the quality and effectiveness of their activities and implementation strength to identify gaps and make necessary course corrections. RAISE applies a standard set of indicators across TCI’s 4 sustainability domains: (1) political and financial commitment, (2) capacity (knowledge) transfer of FP skills, (3) institutionalization of TCI’s high-impact best practices at all levels of the health system, (4) sustained demand through improved attitudes and behaviors toward FP.

TCI identified several predictors of sustainability that helped inform its approach to measuring local government progress toward graduation and greater self-reliance.

Adapted to the context of the health systems, each of these domains is accorded a maximum score. Based on the results of the self-assessment, an overall score is generated, which RAISE workshop participants then validate using contraceptive uptake data, project records, MSC stories, policy documents, budgets, and expenditure reports. Through discussion of these data, participants work to reach a consensus on scores, which map to different levels of capacity and sustainability (high, moderate, basic, and low). Local health management teams and TCI use RAISE results to track city progress and readiness to graduate and provide inputs into actions required to achieve performance milestones. Quarterly RAISE assessments also provide immediate feedback on what is working well and priorities for coaching support. By the third quarter, local governments typically assume full leadership of the RAISE workshops to monitor their progress.^27^

Through June 2021, 39 governments have graduated from full TCI support. TCI is monitoring the performance of the graduated cities by continuing to track RAISE scores and contraceptive uptake, which will inform refinements to its coaching approach and time frames of support. TCI also continues to monitor whether interventions are covered in upcoming government budget cycles.

After graduation, cities continue RAISE assessments and avail TCI-U and community of practice resources and coaching by request for a year or more beyond graduation. Graduated cities become TCI “alumni,” with well-performing graduates recognized for their performance and serving as models for newer TCI cities to emulate. Graduated cities that encounter setbacks can be reengaged and offered support, as appropriate.

**FIGURE 3:**
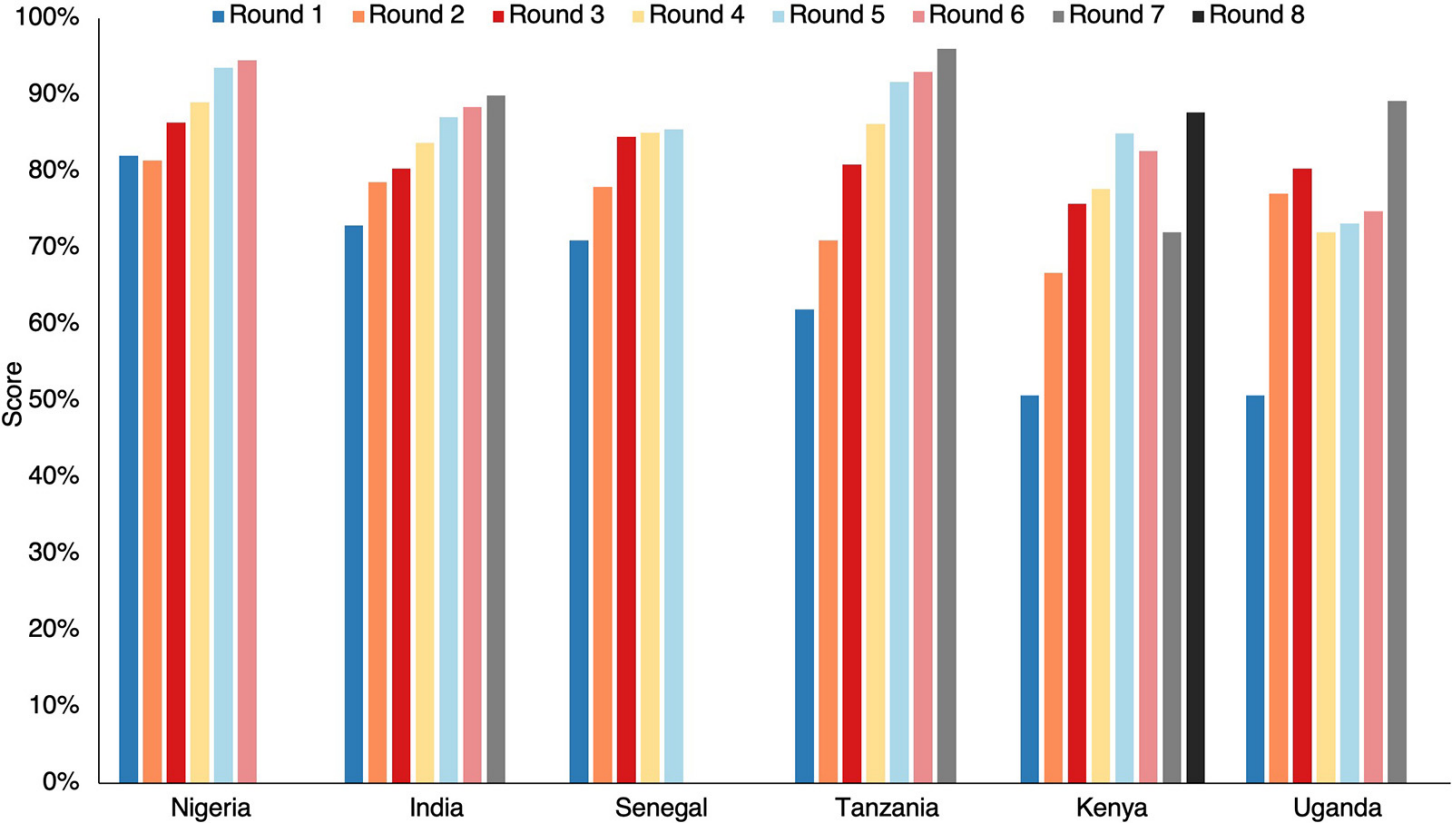
An Average of Graduated Cities' RAISE Scores in 6 Countries Over Time^a^ Abbreviation: RAISE, Reflection and Action to Improve Self-Reliance and Effectiveness. ^a^ The last 1 or 2 rounds occurred postgraduation in India, Kenya, Nigeria, Tanzania, and Uganda. Senegal’s graduated cities had not yet conducted post-graduation RAISE assessments.

Figure 3 shows the average RAISE scores of graduated cities in 6 countries. Even after graduation, RAISE scores (represented in the last 1 or 2 rounds in each of the countries in Figure 3) are generally maintained above 85%, the threshold a city must achieve to graduate. The improved scores demonstrate continued or improved capacity to sustain the interventions.

### Government Political and Financial Commitment

The government’s annual monetary commitment (and spending) is important to affirm government support and is a measurable demonstration of its continued interest and prioritization of FP, given the multitude of other issues needing funding. Government funding is also critical to the ownership and sustainability of the program.

**FIGURE 4:**
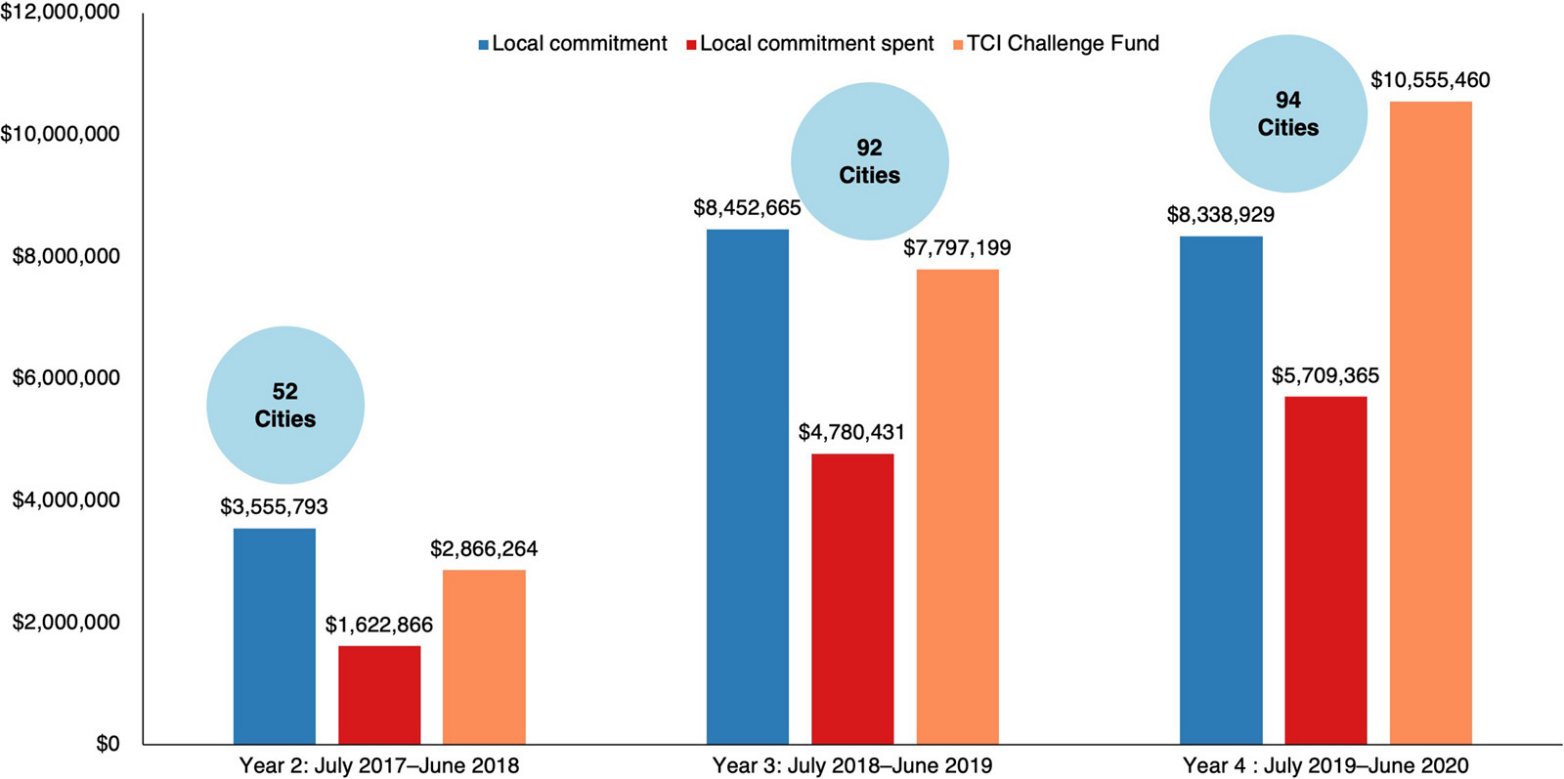
Local Commitments, Local Spending, and the Challenge Fund Allocations Across TCI Hubs (Years 2–4), in USD Abbreviations: TCI, The Challenge Initiative; USD, U.S. dollars.

Through June 2021, local governments committed approximately US$28 million to implement the proven interventions, with a large proportion of these funds contributed by India. Overall government commitments exceeded the amount spent (Figure 4). The percentage of funds spent varies across hubs and cities, with some spending even more than what was committed. In Year 4 (July 2019–June 2020), 70% of committed funds were spent across 4 hubs, with Nigeria (88%) and India (72%) leading the way. Average spending as a proportion of total commitments by local governments across Years 2, 3, and 4 stands at 61%.

Aside from sustainability at the city level, TCI also seeks to secure sustainability of its overall scaling platform by diversifying its donor base and increasing its operational efficiency. Beyond the initial investment of the Gates Foundation, the platform has attracted investments from Bayer AG, Comic Relief, the U.S. Agency for International Development, and private philanthropists with funding for the platform in place through December 2025. TCI will seek to further diversify funding sources in the coming years.

Aside from sustainability at the city level, TCI also seeks to secure sustainability of its overall scaling platform by diversifying its donor base and increasing its operational efficiency.

### Cost-Efficiency

TCI monitors the platform’s cost-efficiency over time by tracking the unit cost per engaged city, million population footprint, and additional FP client. These indicators gauge whether the platform is operating more efficiently over time as it partners with a greater number of cities. In its fifth year, TCI’s cost was US$88,080 per engaged city, US$79,675 per million population footprint, and US$25 per additional FP client. Relative to the unit costs recorded in TCI’s second year, these unit costs have declined by 81%, 76%, and 66%, respectively.

While these measures are critical to track and calculate, the cost-efficiency of achieving the qualitative outcomes for sustainable scale-up is also important though more challenging to track; methods are needed to account for hard-to-quantify contributions to health systems strengthening, sustainability, and diffusion that are delivered as part of the same unit cost. TCI is working to strengthen its cost-efficiency measures to better reflect the value proposition offered by a global scaling platform as well as capture the unit cost for each city and each additional client as the platform evolves to demonstrate economies of scale and scope.

## EXTERNAL PROGRAM REVIEW FINDINGS

In 2020, the Gates Foundation commissioned an external program review to assess TCI’s implementation strength, potential for sustainability, impact, and cost-effectiveness. Supplement 3 summarizes the review’s methods and findings, some of which we describe here.

TCI’s platform offered comprehensive, practical resources to governments to support implementation (TCI-U), installed on-site managerial and technical support through local organizations, and built widespread demand through skillful marketing.

While managerial coaching was generally robust, the strength of coaching on the supply- and demand-side interventions varied across geographies, as did the rollout, intensity, and coverage of these interventions. For example, the review found that AYSRH service provision had been institutionalized in India and there was progress toward institutionalization in East Africa and Nigeria (Box 3).

BOX 3 Excerpts from The Challenge Initiative Program Review Findings Related to Implementation Strength and Sustainability

- It was not always easy to achieve the delicate balance between encouraging ownership (by putting government officials at the helm of coaching cascades) and ensuring facility- and community-level stakeholders feel prepared for implementation (by supplementing government coaching with targeted additional support).
- Since TCI focuses on capacity building and system strengthening rather than direct implementation, impacts on [modern contraceptive prevalence rate] may be slow to appear.

Challenges that impede implementation include difficulties accessing Challenge Fund and government resources, staff turnover, and commodity stockouts.

TCI’s approach enabled the integration of interventions into policies, workplans, and budgets. There is evidence of a “diffusion effect” with nonparticipating governments independently implementing these interventions using their own resources.

The key sustainability risks that cities face postgraduation include maintaining effective coaching, political will, and data-driven decision-making systems.

The key sustainability risks that cities face postgraduation include maintaining effective coaching, political will, and data-driven decision-making systems.

At the start of the program review, an analysis had been conducted of 16 of 79 cities that had been implementing TCI’s approach for 18–24 months. The review found evidence of limited impact in many geographies but also evidence of moderate to large impacts in some countries, particularly in Nigeria and Uganda. The review also found variable evidence of improvement in quality measures (such as reduced commodity stock-outs and provider training), which was large in some geographies, such as India and Kenya, and less in other geographies. More geography-specific results from the program review findings are included in Supplement 3.

The program review findings have significantly informed the design of TCI’s second phase (2021–2025).

## DISCUSSION

In this section, we consider key issues raised in the scaling literature, examine how TCI addresses them, and discuss learnings and challenges.

### What Approaches Build Capacity and Strengthen Government Systems to Aid Scale-Up?

What are effective approaches for building the capacity and strengthening the systems of host organizations to sustainably scale up a proven intervention?

There is strong agreement in the scaling literature that programs need to “begin with the end in mind.”^1^ This entails involving the host organization early and designing and testing an intervention based on a realistic assessment of organizational and environmental constraints, assets, and systems if there is to be a reasonable chance for the new intervention practices to be mainstreamed into government programs.^6^^,^^32^ There is also growing recognition of the need for a meaningful period built into the timeline during which the intermediary organization fully transitions responsibilities and capacities to the host organization.^33^^,^^34^ While there are now many studies that describe best practices for this transition, there are few empirical accounts of how this process is being or has been undertaken.

#### Coaching and Digital Tools

To build sustainable capabilities into systems critical to effectively deliver the FP interventions, TCI’s coaching focuses on finance, service delivery, workforce, data, and leadership and governance, working with existing training and supervisory personnel as master coaches. TCI-U’s digital tools and community of practice facilitate the transfer of capabilities into relevant system areas and have become even more important after the COVID-19 pandemic. As an online tool, TCI-U enabled support to continue amid strict restrictions on movement. Remote learning and coaching will likely continue to play a larger role in the future (Box 4).

BOX 4 Quote on Outcomes Resulting From The Challenge Initiative’s Coaching Support

*With [The Challenge Initiative, TCI’s] support, we’ve taken a much broader approach to urban family planning. TCI initially supported us by demonstrating interventions. It coached us on microlevel analysis of family planning data at the health facilities and [accredited social health activist, ASHA] level also. We learned steps for implementing [high impact practices] and gradually began leading interventions and coaching sessions ourselves. I coached the medical officer in charge, the urban health coordinator, the [Family Planning Logistics Management Information System, FPLMIS] Manager, the [health management information system, HMIS] manager, data entry operators, and others through in-person and group coaching sessions. I have coached my coachee on identifying data errors and inconsistencies, validate data, data analysis, interpretation of data, and fix errors, and resolve issues by formulating action plans. Prior to district level review meetings, I categorize facilities as high-performing, medium-performing, or low-performing in order to present urban family planning HMIS data. This aided our department in making timely course corrections and in planning technical training for service providers on [intrauterine device] and new contraceptives, training of ASHAs and [urban primary health center] staff on FPLMIS online indenting, shifting trained service providers to facilities where service providers were unavailable, timely arrangement of family planning products and equipment at facilities, streamlining ASHA incentive payments and providing coaching support to all workers.* — Priyansh Shrestha, TCI master coach, Divisional Urban Health Consultant, National Urban Health Mission, Kanpur, India

BOX 5 Quote on The Challenge Initiative Coaching Experience

*[The Challenge Initiative, TCI] was like a university because I learned almost everything to make me excel in this position. TCI coached me on how to prepare a standard memo (for [family planning] budget allocation) and follow up on a submitted memo for successful approval. And by God’s grace, I will soon have one of the memos I submitted approved… TCI strategies have helped change behaviors even at the [local government area] and community levels. For instance, [quality improvement teams] has helped unite the facility and the community, which has brought a good synergy in addressing some of the misconceptions and quality issues not addressed before the coming of TCI. The [whole site orientation, WSO] was an eye-opener to all the untrained clinical staff in facilities. The state is currently planning to scale up the WSO to other additional facilities because it has helped address misconceptions spread by some of these non-clinical staff. Looking at the social mobilizer trained specifically on family planning, they have been mobilizing so many clients to the facilities.* —Mrs. Sambo Omar, Family Planning Coordinator, Gombe State, Nigeria (where TCI support began July 2021)

That said, because most graduated cities have had less than 1 year, it remains to be seen the degree to which coaching is sustained by local governments to become an internalized, continuous need within the health system rather than a short-term, externally driven effort that recedes with project closeout. The program review raised this issue, which is being closely monitored and designated as a priority area of learning for TCI. TCI is also exploring how to strengthen the TCI-U platform with enhanced instructional design technology to create greater efficiencies in how coaches transfer technical and managerial capacity (Box 5).

It remains to be seen the degree to which coaching is sustained by local governments to become an internalized, continuous need within the health system rather than a short-term, externally driven effort that recedes with project closeout.

### What Incentives Facilitate the Adoption and Sustained Commitment to an Innovation?

What are effective incentives to introduce the adoption of innovations into public health systems and to sustain the host organization’s commitment to change beyond the period of external support?

The private sector generally has fewer challenges with scaling up novel approaches that have been shown to improve profit margins; competition and profit (often aided by large marketing investments) act as powerful motivators to change the behavior of markets.^25^ The public sector lacks this market motivation and confronts a much greater and more complex set of challenges as it takes on social challenges that the private sector may not see as its primary responsibilities. The literature on scale cites attributes of the innovation as well as the critical role of government leaders, policy champions, an activated adopter community, and other “change agents” that drive people and systems to alter their usual practices and adopt and sustain evidence-based interventions.^8^^,^^11^^,^^13^ Alignment of scale goals with the public sector’s health strategies, policies, and priorities is also crucial for mobilizing support for a change in practices and processes.^24^ Less frequently mentioned—albeit more challenging to demonstrate as potentially powerful incentives to facilitate change processes—is the role of relationships and trust.

#### Funding Commitment

Governments have been highly receptive and even enthusiastic to partner with TCI and lead the ongoing FP intervention scaling process. At any point in time, there are always more cities that have submitted a letter of inquiry than the platform can enroll. Some of the eagerness of local governments to partner with TCI can be attributed to the Challenge Fund’s financial incentives.

Not surprisingly, annual counterpart funding has been impacted by events, both expected (e.g., elections and changes in political leadership) and unforeseen (e.g., COVID-19 pandemic). Aligning program interventions with the health budget planning processes and other existing government policies and workplans mitigated their effect on government contributions in many instances. Ensuring community influencers have the capacity to continuously advocate for FP funding has also been critical to the sustained prioritization of FP investments.

As indicated earlier, the amount of government funds released often fell short of what was committed to TCI, an issue the program review called out as a risk to sustainability. To address this challenge, TCI’s second phase is building on its experience with the Challenge Fund, particularly in Nigeria where nearly 88% of the funds committed for Year 4 were spent, due to an innovative cofinancing model developed by the hub. TCI is testing variations of this cofinancing model in other regions to determine its portability.

To deal with shortfalls in funding that occurred, TCI is testing variations of this cofinancing model in other regions to determine its portability.

To further bolster government spending on high-impact FP approaches, TCI is exploring opportunities to enable “pressure from below” by mobilizing organizations that work closely with local governments (e.g., National Council on Population and Development in Kenya) to increase spending of committed FP funds, as recommended by the program review. Already, TCI has built on URHI’s success in Nigeria working with advocacy core groups, which comprise a wide range of stakeholders (such as influential community and religious leaders) and provide the voice of civil society to ensure government accountability. TCI has strengthened existing or, where necessary, established new advocacy core groups to ensure sustainability of committed funding and an enabling environment for FP services. TCI is also exploring the program review’s recommendation to strengthen or more widely activate nongovernment organizations to increase spending of committed FP funds in other TCI country settings.

#### In-Kind Support

At least as important as the monetary commitment, local governments contribute “in-kind” resources, such as human resources and time to participate in and lead implementation team meetings, quarterly RAISE workshops, and other processes. Local government staff also dedicate time to coaching both as coachees and coaches to their colleagues. These ongoing contributions and demonstrations of commitment may be explicable by the “soft” incentives provided to participating cities. These include the opportunity to lead all levels and stages of their FP and AYSRH program change processes, ongoing professional development, realizing “real-time” evidence of success on measures aligned with local health bureaucratic goals, celebration of successes, and certificates for completing a TCI-U training. These incentives—built on trust and relationships between the hubs and municipal staff and among municipal staff—have proven vital for a government’s ongoing support for the change process.

#### Maintaining Commitment and Capacity

Finally, the logic of embedding effective interventions into institutions, policies, and practices creates push and pull factors. Trust and relationships, although always important, may become less so over time as government mandates as represented in public policy, work plans, and budgets create their own logic for change by transforming incentives, accountabilities, and culture, driving municipal stakeholders to become agents of the change process.^4^ In the shorter term, a key obstacle to any change process is staff attrition. The program review cited the turnover of local government staff as a challenge to TCI’s ongoing success. TCI is currently testing several potential solutions. Developing a critical mass of master coaches that can cascade their knowledge and skills down to other staff within local health systems can help limit the impact of turnover. These coaches can absorb and retain institutional capability for FP program management, with a focus on supportive supervision and scale-up of quality high-impact interventions. TCI has also produced “playbooks” in each hub to detail all the unique hub-specific processes involved in TCI operations to help mitigate the loss of trained coaches.

Developing a critical mass of master coaches that can cascade their knowledge and skills down to other staff within local health systems can help limit the impact of turnover—cited as a challenge to TCI’s ongoing success.

### Can Scaling Be Accelerated?

How can we ensure the right balance between adaptation that is critical to creating effectiveness, acceptability, ownership, and sustainability and standardization that is needed for rapid expansion and vital for generating economies of scale and efficiencies?

The scaling-up literature cautions that achieving and sustaining results at scale takes considerable time. The expectation that an innovation or intervention can be absorbed by the host organization and sustained without external support in only a few years is widely recognized as unfeasible.^13^^,^^25^ Can the process of institutionalization be accelerated? In terms of expansion, how do we move beyond linear rates of expansion to new geographies? Barker describes scale-up as a period of “rapid uptake of the intervention through replication,”^8^ and Bradach asks, “How can we get 100x the impact with only a 2x change in the size of the organization?”^35^ How can replication be conducted rapidly and efficiently to potentially dozens or more host organizations, communities, cities, or districts without compromising local fit and ownership? And what is the right organizational structure for an intermediary platform to drive and manage rapid scale across multiple countries? While the answers to these questions depend on the nature and complexity of the intervention, context, and other factors, there is an opportunity and need to look for pathway efficiencies, especially when the coverage aspiration is multicountry.

#### Local Government Partnerships

To accelerate the institutionalization of FP interventions, TCI structures partnerships with local governments with their engagement and leadership built in from the outset (opting in, design, implementation, and performance monitoring). A supported city moves from onboarding to graduation in 3–4 years, although graduated cities continue to avail TCI-U resources and coaching by request for 1 year or more beyond graduation. To assist this process and make it more efficient, TCI complements its coaching with a system of incentives along with TCI-U’s digital training and access to guidance, learnings, and implementation “know-how.” In addition, TCI continues to work closely with health system staff to embed agreed-upon goals and budgets into policy documents, official guidelines, and even job descriptions. For example, job descriptions for auxiliary nurse midwives in Firozabad in Uttar Pradesh, India, now include attending fixed-day static services—a TCI high-impact intervention—at urban primary health centers to help alleviate chronic staffing shortages.^36^

#### Platform Management Structure

To accelerate expansion, the TCI platform was designed to rapidly scale up proven FP interventions across multiple cities in multiple regions. The standardization of its systems and processes combined with meaningful engagement with local stakeholders and systems allows the platform to create a pipeline of cities moving onto, through, and off the platform without requiring the platform itself to bloat. In this sense, TCI has many features of a franchise model. The platform manager role is akin to a franchisor, with the hubs acting as franchisees. The global platform manager defines and upholds a common set of practices and approaches for the hubs, including an engagement and phasing approach, metrics, coaching standards, and milestones. This standardization of rules across all hubs supports quality, organized learning, and scale efficiencies that aid rapid scale across multiple geographies simultaneously. TCI continues to monitor the degree to which and how the hubs and global platform achieve economies of scale as the platform expands.

### What Measures and Data Sources Support Sustainable Scale-Up?

Contraceptive uptake and coverage are important but insufficient measures for evaluating scale-up success.^32^ Inherent in the concept of sustainable scale-up is strengthened capacity of the host organization to independently implement the innovation and the institutionalization of the change practices into host organization systems and policies. Hence, success measures must also assess sustainability, including system and leadership capabilities and the degree to which they are being transformed to accommodate the new intervention practices.^2^^,^^37^ The cost-efficiency of the scale-up process also needs to be tracked. We should expect to see economies of scale (and learning by doing) over time.

#### Mixed-Methods Approach

TCI employs a mixed-methods approach using both quantitative and qualitative data to systematically capture, analyze, and learn from its programming. To gauge progress along the scaling pathway, a broad set of indicators (Supplement 4) are tracked related to uptake (contraceptive use), coverage, sustainability, and cost-efficiency generated by RAISE, MSC, project records, and HMIS, which is strengthened through coaching.

#### Local Data Sources

The reliance on locally generated data sources significantly lowers the cost of monitoring performance compared to instituting population surveys in all geographies where TCI operates, which is not feasible. As important, service statistics and other local data sources generate data that is timely and relevant to local, including municipal, programs. Existing population surveys such as Demographic and Health Surveys^38^ and Performance Monitoring for Action^39^ are not tailored to capture data on smaller geographical units such as a municipality. Prioritizing sustainability requires putting a premium on existing data systems—those that will continue to be used once cities graduate and the intermediary (and donor funds) are no longer available.

The reliance on locally generated data sources significantly lowers the cost of monitoring performance compared to instituting population surveys in all geographies where TCI operates.

### Can Donor Norms Be Transformed to Finance Scale-Up?

How can programs avoid the “stagnation chasm” that, due to lack of funding, often besets social innovations before they have a chance to scale?^10^ Investors (including donors) are more willing to back a new product launch or test an innovation undertaken on a small scale than an intervention that has already been successfully piloted. This is the case for a variety of reasons, including the often-unfulfilled expectation that governments will scale up approaches that have been shown to work.^2^^,^^4^^,^^10^^,^^35^

#### Long-Term Planning

As with any intermediary scaling platform, fundraising is a critical function. Since 2016, TCI has successfully obtained significant funding from multiple donors, with new commitments secured through 2025. A key lesson from this success, which followed considerable effort, is the importance of assembling a longer-term resource plan with a diverse array of donors, including nontraditional donors and private sector partners.

#### Leverage Other Donors

To motivate donors to make long-term investments in scale-up, a multidonor strategy may require a “piecemeal” approach to funding, with different donors taking on different platform components or countries aligned with their strategies and geographic priorities. Donors often find attractive the opportunity to leverage other donor funds on behalf of their own strategies. Doing so facilitates the “business case” they need to make internally to their organizations for contributing to a partnered effort to scale an innovation that may have been piloted with other donor funding. Partnerships may appeal to donors for other reasons: they may help derisk an ambitious investment and provide an opportunity to participate for even small donors who would not otherwise be able to underwrite the large-scale rollout of programs with potentially large-scale impact.

However, the downside for the intermediary organization of a patchwork approach to funding is resource uncertainty and increased transaction costs in terms of reporting burden, aligning funding cycles and outcomes, conflicting priorities and reporting requirements, and other coordination challenges. Ultimately, the development community, and particularly donors, must work toward a philanthropic norm change that recognizes the value and need to support the longer-term requirements of taking an innovation to scale beyond the common 3- to 5-year project cycle.

## CONCLUSION

There is a growing interest in and awareness of the need for a better understanding of successful models for taking effective interventions to scale to reach underserved communities and meet the Sustainable Development Goals. The necessity to scale up health interventions for the urban poor is particularly acute given the rapid pace of growth and gap in our understanding of how best to meet their needs, including for contraceptives, at scale in a sustainable way.

TCI locates itself squarely within the wider movement to elevate the importance of local leadership and ownership in development efforts as a means to support sustainability and shift development decisions to local governments and communities affected by them.

We described the TCI model and described how its design has been responsive to the key challenges typically faced by development programs with aspirations to scale. TCI is still a relatively young platform and in learning mode. Building on the lessons from URHI as well as successful scaling programs elsewhere, the TCI platform has taken a “learning by doing” approach to build its regional and global platforms for catalyzing rapid scale-up of proven interventions. Its success to date lies in putting a premium on local government ownership, including local control over implementation and meaningful involvement of key local stakeholders throughout the entire change process. Other key factors for success include the availability of donor funding to support platform operations and incentivize government participation, attention to local data sources for program monitoring and learning, and its “fit-for-purpose” organizational and management structure as an intermediary organization supporting scale-up across multiple countries.

Success going forward will depend to a great extent on whether TCI can learn from and overcome some of the greatest challenges to scaling, including staff turnover; “voltage drops” in contraceptive uptake and loss of momentum toward institutionalization and capacity building postgrad-uation; and challenges securing funding to support sustainable scale-up in new geographies.

## Supplementary Material

GHSP-D-22-00167-Supplements.pdf

## Notes

**Peer Reviewed**

**First published online:** November 14, 2022.
