# Supplementary material for: A Platform for Sustainable Scale: The Challenge Initiative’s Innovative Approach to Scaling Proven Interventions
Source: Glob Health Sci Pract. 2024 May 21;12(Suppl 2):e2200167. doi: 10.9745/GHSP-D-22-00167 (PMC11111104; doi:10.9745/GHSP-D-22-00167)
Supplement: GHSP-D-22-00167-Supplements.pdf [file GHSP-D-22-00167-Supplements.pdf]

Supplement to: Finkle C, Martin K, Salas I, et al. A platform for sustainable scale: The Challenge Initiative's innovative approach to scaling proven interventions. *Glob Health Sci Pract*. 2023;11(Suppl 1):e2200167. https://doi.org/10.9745/GHSP-D-22-00167

Supplement 1
TCI's Coaching Model

The Challenge Initiative (TCI) uses a locally owned and implemented model of coaching with a systematic yet flexible process to build capacity and strengthen health systems. TCI coaching is premised on the principle that governments should be engaged as lead partners of development interventions. TCI coaches serve as resources and technical advisors to city teams to activate existing systems and solve problems.

TCI coaching is conducted by local and regional master coaches who cascade knowledge and skills in implementing the evidence-based interventions and tools to municipal health personnel and managers to strengthen local health systems.

Initially, TCI hub staff serve as master coaches but transfer this role to local experts with strong family planning and AYSRH experience. In consultation with TCI, city teams identify managers and implementers for training as TCI coaches relevant to specific proven interventions. For service delivery interventions, for example, TCI and local government counterparts review the list of those who currently provide supportive supervision and are considered master trainers of the government as a good starting point for resource persons to be trained as TCI coaches within the public health system. Once coached by TCI, they then cascade down what they learned to leadership, lower level managers, and implementers. Coachees may include political leaders, ministry of health leadership, managers (Medical Officers-In-Charge, Nurses-In-Charge/Matrons, FP/RH coordinators, family planning officers, urban health officers, health educators, monitoring and evaluation officers, and adolescent health officers), and implementers (frontline workers such as family planning, health district, facility-based personnel, and community health workers). Thus far, more than 3,300 coaches have been trained across the platform's hubs.

TCI's approach goes beyond technical coaching on proven interventions. TCI also coaches on effective management, such as helping cities improve coordination between local family planning actors, engaging with private facilities, facilitating community accountability, supporting cities to use data for measuring progress and course corrections, and improving processes on planning, budgeting, and finance.

Coaching typically starts at high intensity, but gradually tapers off as implementation progresses and local governments gain confidence and skills – what TCI refers to as its "Lead-Assist-Observe" coaching model (Fig. 1). After about three years, a local government "graduates" to a coaching-on-demand model and less financial support from TCI based on their performance, which is assessed throughout their engagement with TCI using the RAISE tool (Supplement 2).

Supplement to: Finkle C, Martin K, Salas I, et al. A platform for sustainable scale: The Challenge Initiative's innovative approach to scaling proven interventions. *Glob Health Sci Pract*. 2023;11(Suppl 1):e2200167. https://doi.org/10.9745/GHSP-D-22-00167

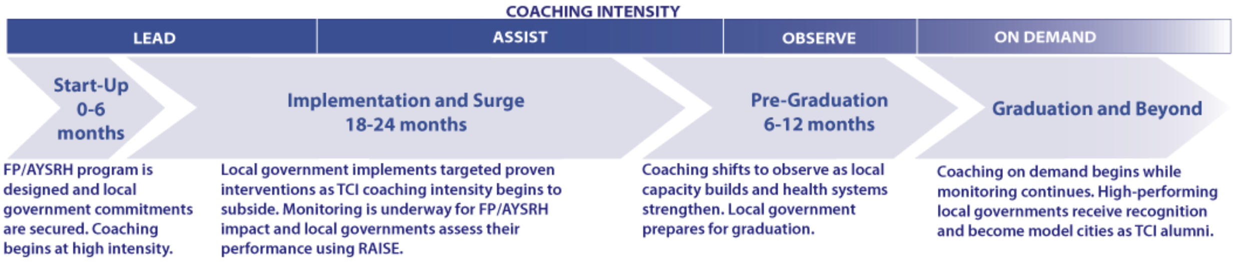

Figure S1: TCI's lead-assist-observe coaching model alongside local government engagement timeline.

Supplement to: Finkle C, Martin K, Salas I, et al. A platform for sustainable scale: The Challenge Initiative's innovative approach to scaling proven interventions. *Glob Health Sci Pract*. 2023;11(Suppl 1):e2200167. https://doi.org/10.9745/GHSP-D-22-00167

Supplement 2. Preliminary Analysis of Family Planning Trends Post-Graduation Through February 2022

TCI continues to monitor the contraceptive uptake trends in graduated cities. In April 2022, TCI conducted some preliminary analysis of service statistics data in the first 48 graduated cities to investigate various questions of interest. Table 1 presents these questions as well as what TCI found.

Table 1: Preliminary analysis of contraceptive uptake in graduated cities

**Preliminary analysis of contraceptive uptake in graduated cities**	
**Question 1**<br><br>How many additional FP clients are coming from graduated cities?	TCI noted that from the 2.32 million additional FP clients since TCI implementation, a total of **218,439 additional FP clients** were coming from the 48 cities since they graduated from TCI. The breakdown by hub is shown below.
**Question 2**<br><br>Are the graduated cities still showing positive contraceptive uptake since graduation?	Based on a subset of 10 cities with at least 12 months since they graduated from TCI support, TCI noted that **9 out of 10 cities (90%)** still showed a positive number of additional FP clients since graduation. See data in Table 2.
**Question 3**<br><br>Are the graduated cities showing an increasing rate of contraceptive uptake since graduation?	Based on a subset of 10 cities with at least 12 months since they graduated from TCI support, TCI noted that **7 out of 10 cities (70%)** showed an increasing annualized rate in the number of additional FP clients comparing the full period after graduation to the one-year period before graduation. See data in Table 2.
**Question 4**<br><br>Did the graduated cities achieve the NCU target of 1.6 per 100 WRA? This target was set at the beginning of the bridge period.	Based on a subset of 10 cities with at least 12 months since they graduated from TCI support, TCI noted that **8 out of 10 cities (80%)** achieved the 1.6 per 100 WRA NCU target set at the beginning of the bridge period. See data in Table 2.

**Graduated cities in TCI hubs**	**Question 1: Additional FP clients since TCI graduation of each city**
East Africa, 20 cities	135,182
FWA, 2 cities	1,293
India, 21 cities	43,073
Nigeria, 5 states	38,891
All hubs	218,439

Supplement to: Finkle C, Martin K, Salas I, et al. A platform for sustainable scale: The Challenge Initiative's innovative approach to scaling proven interventions. *Glob Health Sci Pract*. 2023;11(Suppl 1):e2200167. https://doi.org/10.9745/GHSP-D-22-00167

Table 2: Number of additional FP clients in cities that have graduated for at least 12 months

Hub	Graduated city	Months since graduation	Additional FP clients one year before graduation	Question 2: Additional FP clients since graduation (full period)	Additional FP clients since graduation (annualized)	Question 3: Change in additional FP clients (annualized since grad minus before grad)	Question 4: NCU after graduation (per 100 WRA)
East Africa	Kericho	21	6,834	18,312	10,301	3,467	3.98
	Uasin Gishu	21	8,000	10,029	5,641	-2,358	1.69
	Ilala	21	19,960	66,938	37,653	17,693	10.15
	Temeke	21	15,672	14,581	8,202	-7,470	2.24
	Busia	21	2,253	-2,002	-1,126	-3,380	-1.15
India	Amroha	14	392	2,775	2,345	1,954	4.05
	Faizabad	14	1,412	2,384	2,015	603	4.18
	Ghaziabad	14	-4,422	3,367	2,845	7,268	0.59
	Muzaffarnagar	14	1,360	7,520	6,355	4,995	5.54
	Noida	14	-1,866	5,977	5,051	6,917	2.72

Note: Graduated cities from Madhya Pradesh and Odisha, India, were excluded from the analysis due to a lack of access to HMIS data in these states beginning September 2020.

Results from answering the four different questions showed positive signals around contraceptive uptake after cities graduated from full TCI support. TCI will continue to monitor HMIS data from its graduated cities – especially as more cities join this analytic cohort – and triangulate it with signals from other data sources. This serves as a great opportunity for TCI to investigate whether uptake of FP services can be sustained even without full TCI support. Findings and learnings from this can also be an important contribution of TCI to the field.

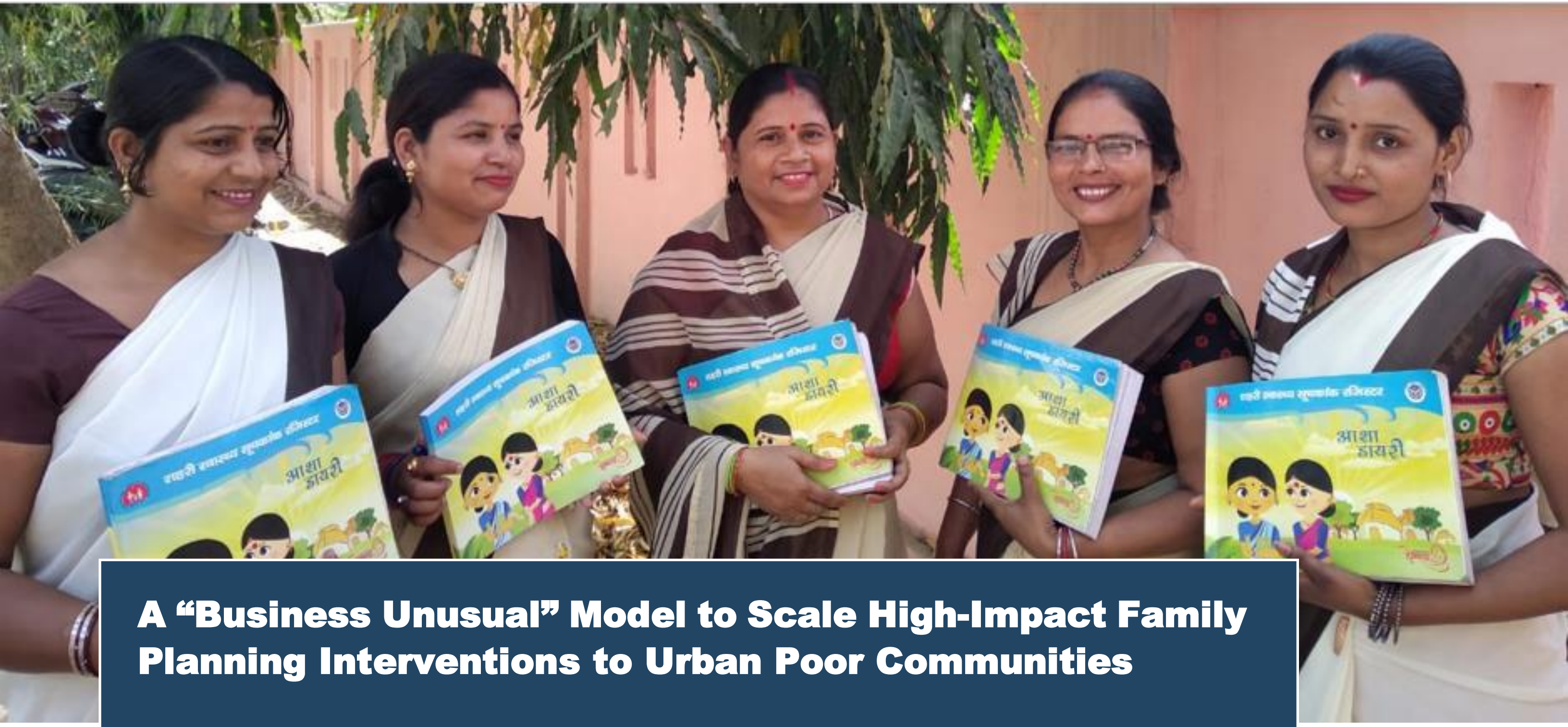

A "Business Unusual" Model to Scale High-Impact Family Planning Interventions to Urban Poor Communities

Summary Findings from the Program Review of The Challenge Initiative

June 2021

More than half of the world's population lives in urban areas, a proportion that is expected to grow to two-thirds by 2050 (United Nations 2018). Ninety percent of that urbanization will occur in Africa and Asia, where the majority of urban residents live in poor and often informal settlements. Health systems are not keeping pace with this dramatic urban growth, and the sexual and reproductive health risks for the urban poor are severe, including high rates of unwanted pregnancies, sexually transmitted infections, and poor maternal and child health outcomes. The Bill & Melinda Gates Foundation launched The Challenge Initiative (TCI) to scale proven solutions to these challenges. Established in 2017, TCI sought to catalyze the rapid adoption and scale-up of high-impact interventions (HIIs) focused on family planning (FP) and adolescent sexual and reproductive health (AYSRH) in urban areas across the globe. TCI tests a "business unusual" approach, whereby interested local governments apply for TCI support and commit to contributing a portion of the funding needed to adapt and implement HIIs in their geography. To support implementation, they receive a range of supports from TCI, including detailed toolkits, tailored coaching, and access to a community of practice. Three years after program launch, the foundation commissioned Mathematica and Avenir Health to conduct a program review to assess the initiative's implementation strength, potential for sustainability, impact, and cost-effectiveness. This brief summarizes the key findings of the program review.

Overview of The Challenge Initiative

In 2010, the Bill & Melinda Gates Foundation launched the Urban Reproductive Health Initiative (URHI) to identify solutions to address the family planning (FP) needs of the urban poor. URHI demonstrated the effectiveness of a range of supply- and demand-side interventions in improving the accessibility, quality, and use of FP services. To catalyze the scale-up of these high-impact interventions (HIIs), the foundation created The Challenge Initiative (TCI) in 2017 (Figure 1). TCI deploys a "business unusual" model, which at its core entails putting local governments in the driver's seat. Geographies (including cities, counties, districts, and states) with health systems ready and willing to make strides in the FP space commit their own funds and resources to participating in the initiative and adapting and implementing proven solutions to key FP challenges. A diverse range of HIIs is available to local governments. They include interventions to improve management of FP programming, support advocacy to increase funding for FP, improve service delivery, and generate demand. Starting in 2018, local governments could also elect to "layer" on programming to support adolescent and youth sexual and reproductive health (AYSRH). To effectively implement these interventions, local governments received a range of supports, collectively called TCI-University (or "TCI-U).

These include:

- Toolkits with detailed protocols, job aids, manuals, and more to guide implementation of the HIIs
- Coaching to build the managerial and technical capacity of local government officials, service providers and community health workers (CHWs), and other community actors
- A community of practice (CoP) to share learnings and experiences (which consists of a global as well as regional groups on the web and WhatsApp)

Geographies participating in TCI also have access to the Challenge Fund, a pool of funds used to supplement the resources governments have committed.

TCI is overseen by the Bill & Melinda Gates Institute for Population and Reproductive Health at Johns Hopkins University ("the Gates Institute"), which plays a central "aggregator" role, assuming overarching strategic and management responsibilities for the initiative. It supports and coordinates with accelerator hubs providing regional support in Nigeria, East Africa, Francophone West Africa (FWA), and India. The hubs are led by Johns Hopkins Center for Communication Programs, Jhpiego, IntraHealth International, and Population Services International.

Since 2017, TCI has grown to 94 geographies in 10 countries (Benin, Burkina Faso, Cote d'Ivoire, India, Kenya, Niger, Nigeria, Senegal, Tanzania, and Uganda).[1] These geographies leverage TCI-U supports and the Challenge Fund to undertake the "TCI journey", which entails the following phases:

- **Self-Select and Commit phase (6 months):** Local governments accepted as participants conduct landscape and gap analyses to understand the status quo and key needs relating to FP and reproductive health (RH). They map their needs to suitable HIIs in the suite of interventions offered by TCI, adapt these to their context, and develop a program design and plan. Following these visioning and planning efforts, government stakeholders work with TCI to determine the budget (including the resources that will be

FIGURE 1. TCI: Key actors, core components, and pathway to impact

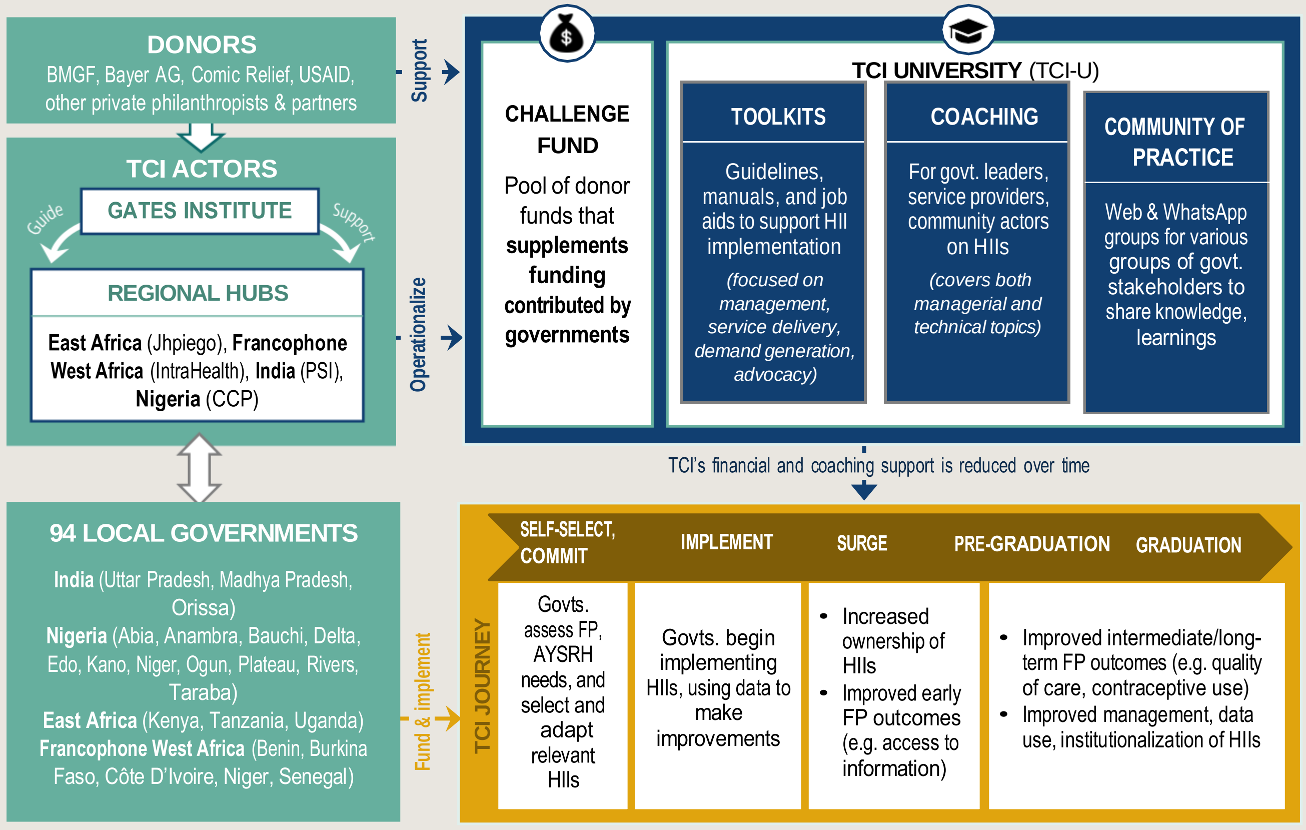

[1] This tally includes geographies in the implementation phase at the end of June 2020, when the program review began. Six additional geographies were in the EOI phase at this time. The number of implementing geographies has grown to 109 (as of March 2021).

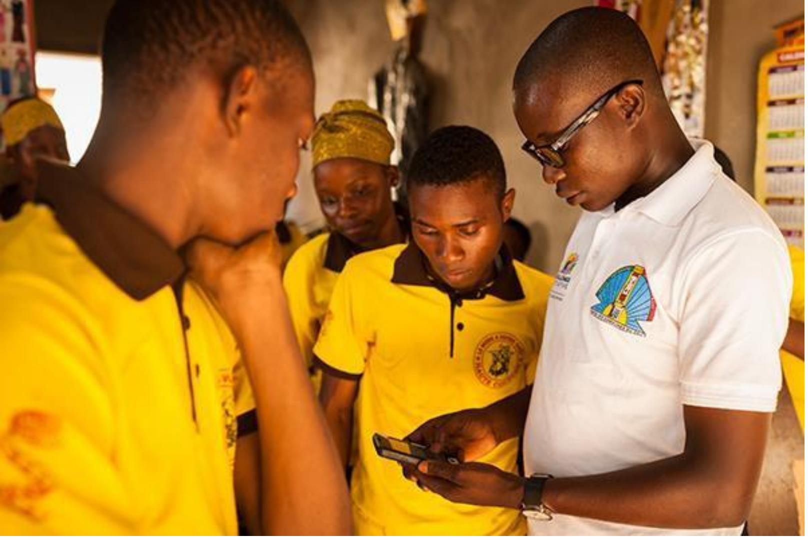

received from the Challenge Fund as well as the resources they will commit themselves).

- **Implement phase (9-12 months):** Local government staff receive coaching from TCI that helps build their understanding and knowledge of the selected HIIs, as well as their leadership and management skills. They then begin implementing supply- and demand-side HIIs, leveraging the TCI-U toolkits. During implementation, they gather and review data to assess their progress and make needed adjustments.

- **Surge (9-12 months):** TCI's vision is that ongoing implementation of HIIs will yield early signals of success and shifts in key outcomes, which will, in turn, engender greater confidence in and ownership of the interventions. Accordingly, government staff expand and refine implementation, drawing on the CoP to learn from other geographies, as well as ongoing coaching (which is slightly less intensive than in the prior two phases). Implementation successes in this phase will ideally also translate to the beginnings of critical sustainability and scale-up outcomes, including strengthened leadership around FP/RH, increases in dedicated funding for FP, improved quality of service statistics and use of data for decision-making, integration of HIIs into policy, and diffusion of HIIs to non-TCI geographies.

- **Pre-graduation and graduation (12 months):** As local governments near the end of the TCI journey, key FP and AYSRH outcomes, as well as the sustainability and scale-up outcomes mentioned above, should be improving. In alignment with this progress, and greater capacity, ownership, and independence on the government side, coaching support is provided on an as-needed basis at this juncture. To assess readiness for graduation, TCI draws on data from a sustainability self-assessment from local governments known as RAISE, as well as other data. Graduation takes place over a period of six months to a year, while TCI monitors the governments to ensure they can maintain impacts on target outcomes without TCI support.

Methods

Implementation Strength and Sustainability

As part of our efforts to gauge implementation strength, we, the program review team, sought to assess (1) the rollout of the TCI platform, (2) uptake and perceptions of TCI supports, (3) the strength of HIIs to improve FP program management and facilitate improved advocacy around FP, and (4) the intensity, coverage, and quality of service delivery and demand generation of HIIs implemented on the ground. We also sought to gain insight into the operational effectiveness of the initiative, particularly the value-add of the aggregator, the role of the hubs, and the strength of the initiative's Program Evidence, Analytics, Research, and Learning (PEARL) function. As part of our efforts to assess sustainability, we sought to gain insight into the extent to which there was ownership of the HIIs among government officials, the intensity of support provided by TCI, the integration of HIIs into policies and strategic plans, and the diffusion of HIIs into non-TCI geographies.

To gather data on these topics, we conducted "deep dives" in 17 TCI geographies across six countries (India, Burkina Faso, Senegal, Kenya, Uganda, and Nigeria). These geographies were selected to represent the diversity of contexts in which TCI operates. As part of the deep dives we conducted 196 key informant interviews—with government officials at various levels, service providers, community health workers and other community actors, and a range of TCI stakeholders (including hub and Gates Institute staff). We also drew on several data sources shared by TCI, including annual TCI-U user assessment surveys, project record data tracking the implementation of service delivery and demand generation HIIs, and data from TCI's RAISE tool, which measures progress along the TCI journey to self-reliance.

Impact

Our approach to measuring TCI's impacts involves constructing geography-specific synthetic controls to estimate what would have happened to contraceptive coverage in the absence of TCI. These synthetic controls are weighted combinations of non-TCI geographies that are similar to each TCI geography in terms of pre-TCI outcome trajectories and characteristics related to the outcome. This approach accounts for underlying differences between TCI geographies and non-TCI geographies—which is especially important given that geographies self-select into the program. We estimate impacts on two measures of

contraceptive coverage, estimated modern use (EMU)[2] and couple years of protections (CYPs)[3] per 1,000 WRA, constructed using service statistics from HMIS data following the Track20 methodology. In geographies for which it is not possible to construct a synthetic control, we examine trends in contraceptive coverage in TCI geographies relative to trends in geographies with similar pre-TCI mCPR. In the complex, multipartner programming landscape in which TCI operates, the impact estimates and trends can be interpreted as measuring whether TCI affected FP outcomes over and above other "business as usual" FP programming in each country.

Our impact and trend analyses of contraceptive coverage focused on geographies where TCI had been implementing for at least 18 months or were in the graduation or pre-graduation phase as of December 2019 and that had sufficient data quality to reliably interpret trends. We examined 59 TCI geographies from four countries—India (Madhya Pradesh and Uttar Pradesh), Kenya, Uganda, and Nigeria—representing all TCI hubs except FWA, where we were unable to access data in time for the program review. In the analyses, these geographies are covered by 42 administrative units—districts in India, counties in Kenya, districts in Uganda, and states in Nigeria. This approach allowed us to consistently define and identify TCI and non-TCI geographies in each country in the HMIS data for the analyses.

To examine changes in other program outcomes among TCI's Key Performance Indicators that are not available in HMIS data—particularly demand for and quality of FP services—we conduct a post-TCI trend analysis drawing on survey data from PMA Agile[4] in nine geographies across four countries: India, Burkina Faso, Kenya, and Nigeria. We triangulate our findings with information on the context, including baseline contraceptive prevalence, as well as findings on implementation to shed light on variation in FP use and proximal outcomes related to FP use across TCI geographies.

Cost-efficiency and cost-effectiveness

To understand TCI's cost drivers we utilize a top-down costing approach to estimate expenditures at the hub and geography level, including all funding sources, triangulating across multiple data sources as available, and supplemented

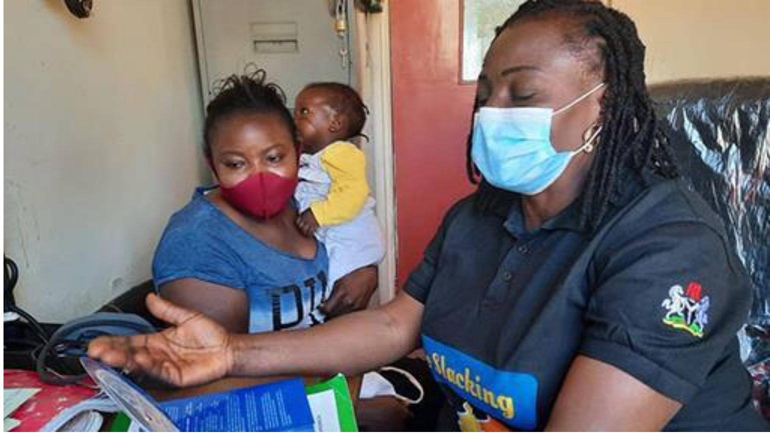

with information from key informant interviews (KIIs) with financial staff at the Gates Institute and the hubs. We explored two measures of cost-efficiency: cost per additional users of modern contraception and CYPs derived based on the impact analysis results. We also calculated incremental cost effectiveness ratios for each geography included in the synthetic control analysis, for modeled unintended pregnancies averted, unsafe abortions averted, and maternal deaths averted, all based on the EMU impact estimates. Finally, we test for economies of scale and learning-by-doing effects by estimating a cost function.

Findings: Implementation Strength and Sustainability

INITIATIVE LAUNCH: **Initiative partners stood up an ambitious platform to facilitate scale-up of HIIs—one that offered comprehensive, practical resources to governments to support implementation, installed on-site managerial and technical support through local organizations, and built widespread demand through skillful marketing.** In developing 37 global or cross-cutting toolkits and 82 region-specific toolkits of step-by-step protocols, technical manuals, job aids, and more—TCI created a vital resource for implementation of HIIs in its target geographies, one that is also a global good. TCI also created a host of useful self-reliance tools, including templates and guidelines for governments to systematically assess their FP landscape, gauge their needs and map them to suitable interventions, and use data to drive action. To match this rich knowledge bank with in-person support, the Gates Institute operationalized four active regional hubs, working hard to build their capacity to coach local government stakeholders on management, advocacy, and supply- and demand-side HIIs, and shift their orientation from "doing" to coaching or supporting. The hubs, which

[2] EMU is measured as the share of the population of WRA estimated to be using a modern contraceptive method, based on data reported in the HMIS about the number of visits, users, or commodities distributed to clients. EMU is the primary outcome of the impact assessment and trend analysis because changes in EMU can be interpreted as changes in contraceptive coverage. Although it is not possible to measure mCPR using FP service statistics, EMU aims to approximate trends in mCPR and is a complementary indicator that can help track changes in contraceptive use between surveys. EMU levels cannot be directly interpreted or compared to mCPR; however, trends in EMU indicate whether contraceptive coverage is increasing or decreasing over time.

[3] CYP is a metric that converts FP commodities from service statistics into a single metric that estimates the aggregate amount of protection (in years) provided by the volume of FP services distributed in a set time period (a month, year, or other unit).

[4] PMA Agile is a data monitoring and evaluation system that collects FP and contraceptive provision data from health facilities and clients in some TCI countries.

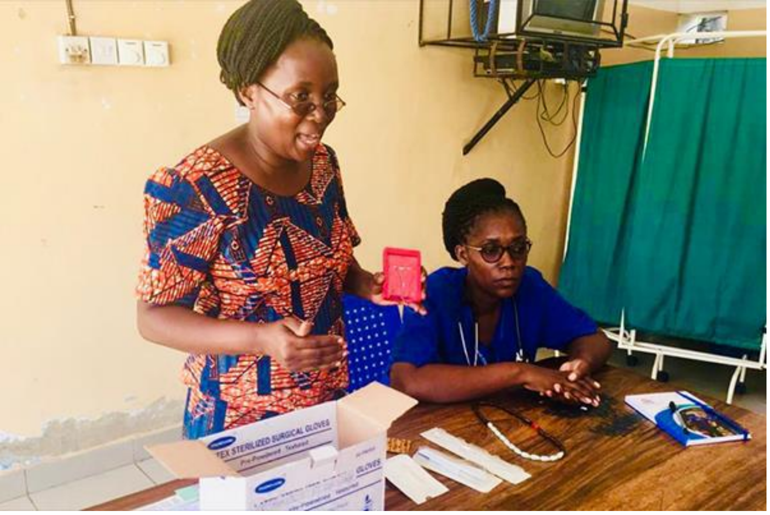

were managed by well-respected organizations with deep contextual knowledge, were very successful in building widespread demand for the initiative. Through their skilled, multipronged marketing, the initiative has scaled rapidly, to 94 geographies by the end of Year 4.

USE OF TCI SUPPORTS: Use of the TCI-U toolkits is high, and managerial coaching has been largely strong, but coaching on supply- and demand-side HIIs is variable across geographies and the use of the CoP was mixed. TCI-U annual user survey data show that use of the site is frequent, and our interviewees indicate they find the toolkits clear and comprehensive (a "one-stop shop"). Coaching falls into two main categories—(1) managerial coaching provided by hubs to senior government officials responsible for overseeing implementation of HIIs, and (2) technical coaching on supply- and demand-side HIIs (which involves hub staff coaching senior government officials, who in turn cascade coaching down to service providers and CHWs and other community actors, with some support at different levels of the cascade from the hubs). The managerial coaching received mostly strong reviews, especially the focus on continuous learning and improvement and the easy access to hub coaches. The coaching on supply- and demand-side HIIs appears to vary substantially across hubs in terms of intensity, which is expected, given TCI's vision was for coaching to be tailored to each geography depending on its needs. While there were several positive perceptions of this support, there were also some requests for additional coaching. It was not always easy to achieve the delicate balance between encouraging ownership (by putting government officials at the helm of coaching cascades), and ensuring facility- and community-level stakeholders feel prepared for implementation (by supplementing government coaching with targeted additional support). As for CoP groups, connectivity challenges, language barriers, and time constraints did affect access. However, those who used CoP groups found them helpful for sharing questions and learning from other geographies.

ADVOCACY: TCI brought about a range of impressive policy and financing wins that enabled implementation of HIIs and also strengthened prospects for sustainability. TCI conducted intensive advocacy to local governments, and also mobilized FP champions and advocacy bodies to engage with these stakeholders, to ensure adequate funding for FP programming and establish other infrastructural and institutional supports. Key achievements include the introduction of dedicated budget lines for FP in 10 Nigerian states and in urban programme implementation plans (PIPs) in three Indian states. Funding for FP was also integrated into annual operational/work plans in a range of our deep dive geographies—in Kenya, Uganda, and Burkina Faso. In addition to these financing wins, new staff positions were created in Kenya and Senegal. In India, the basket of contraceptive method choices was expanded to include IUCDs and injectables.

MANAGEMENT CAPACITY: There was some improvement in government capacity to manage HIIs and FP programming more broadly, but there is room to improve. TCI has helped to establish government-led committees to manage the implementation of HIIs in each of its target geographies. Some are very active and have helped to strengthen key health systems functions. They meet regularly, use data to track service delivery and take corrective action, engage all relevant health, finance, and other stakeholders to reduce implementation roadblocks, and coordinate across non-government partners to avoid duplication. Others, however, have met only sporadically and appear somewhat disengaged. This is due to high levels of turnover among government officials (discussed further below) and competing demands on staff time.

IMPLEMENTATION: Rollout, intensity, and coverage of key supply- and demand-side HIIs vary substantially across hubs. TCI's project record data on the supply- and demand-side HIIs did not uniformly monitor all the interventions, and for the interventions tracked, did not always measure the rollout of capacity-building or the intensity and reach of interventions. Still, using the data shared and triangulating with qualitative data where possible, we sought to develop an implementation snapshot. Broadly, between 80 and 100 percent of targeted facilities have implemented some type of supply-side intervention that involves having a range of health workers at facilities provide FP information, counseling, and services. Regional variations of fixed-day services have been rolled out relatively widely by each hub, but the frequency of these activities is variable. AYSRH service provision is widely institutionalized in India, and there is progress in East Africa and Nigeria. Demand-generation activities are highly diverse across hubs, and some are conducted widely and frequently. But these activities are sometimes challenged by low motivation/desire for financial remuneration among CHWs/community agents.

IMPLEMENTATION: Key factors inhibiting implementation included challenges in accessing funds, staff turnover, and commodity stockouts. The milestones

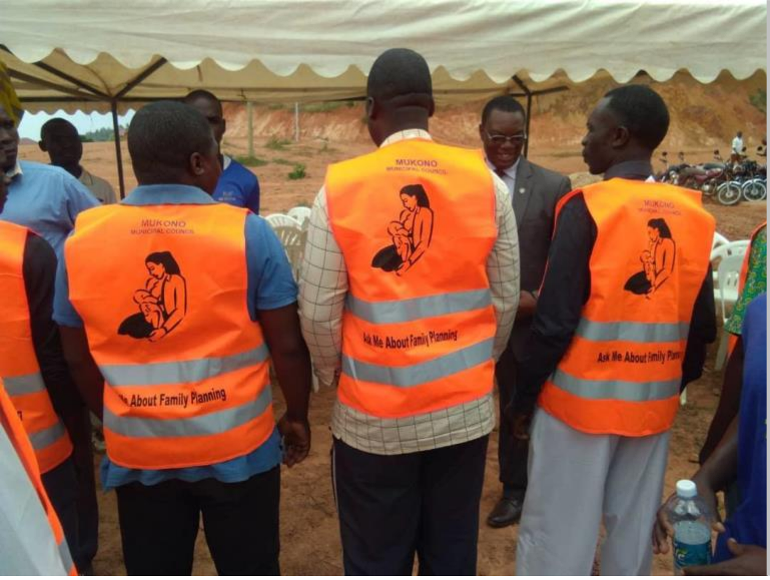

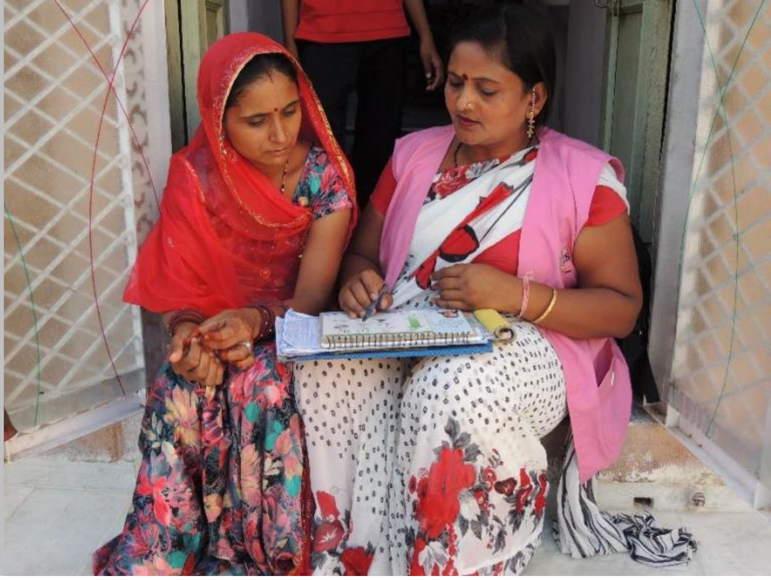

system (a TCI system that attaches disbursement of financial resources to implementation milestones) was confusing to government stakeholders and sometimes misaligned with implementation calendars, which caused implementation delays. A range of bureaucratic issues also delayed access to funds committed by governments. Widespread staff attrition and shortages have led to loss of ownership and momentum, and stops and starts in implementation, as did stock-outs, which were linked to a range of procurement and supply chain issues.

SUSTAINABILITY: **TCI has been successful in pushing forward institutionalization and diffusion of HIIs.** Hubs successfully advocated for the integration of TCI interventions into policies as well as government work plans and budgets by highlighting early successes of these interventions on the ground, connecting interventions with pre-existing policy priorities, and leveraging political will around FP. In addition to ensuring the sustainability of HIIs in the target geographies in the long term, this has enabled diffusion to non-TCI facilities. Some examples of these institutionalization successes are provided below (the full report lists all examples of policy adoption uncovered in our deep dives):

- The Uttar Pradesh government endorsing all nine TCI Classic HIIs in India for scale-up across the state
- Niger state in Nigeria integrating the Whole Site Orientation, Quality Improvement Team, and inreach HIIs into its Annual Operational Plan
- The Universal Referral (or "ISBC") HII being integrated into the health system at the regional level in Senegal
- Key HIIs (in-reaches, outreaches, commodity forecasting, AY services) being included in the annual workplans of Kilifi and Kericho counties in Kenya

There is also demand-driven replication taking place, i.e. geographies that are not participating in TCI see the positive results of TCI interventions in other regions and decide to implement these solutions independently. Non-government partners have also seen and bought into TCI interventions and selected to adopt them in their target geographies.

SUSTAINABILITY: **There is still uncertainty about whether or not certain elements of the TCI approach will be sustained when TCI exits geographies.** TCI geographies are just starting to graduate, so we will have a clearer sense in the future about whether key elements of TCI can be sustained without ongoing support from the hubs. However, our current analysis allows for some diagnosis of the potential for sustainability. Strong cohorts of government coaches will likely continue in some, but not all geographies, and the case is similar for the program implementation committees established by TCI. Due to TCI's persistent outreach and advocacy interventions, the policy environment around FP is strong in many of the geographies, and substantial funding has been committed to FP. However, this political will and dedicated funding runs the risk of flagging in areas where advocacy/watchdog institutions have not been activated. The management and data-driven decision-making systems and processes that have been established through TCI's support may not continue after a geography graduates from TCI, given they are often reliant on TCI funding and logistical support. Cohorts of coaches exist but they may need incentives or motivation to continue doing their work and reinforcing best practices. TCI is learning about how to reduce these risks as increasingly more geographies graduate from the initiative.

MLE: **TCI leveraged HMIS data to construct a proxy measure for mCPR that allowed it to monitor progress on FP outcomes on a near real-time basis, but the approach used to construct the outcome measure did not fully address underlying challenges with HMIS data quality.** TCI's efforts demonstrate the potential for using HMIS data for real-time monitoring, but their analysis and interpretation did not take into account the quality of data and contextual factors that affected reporting.

MLE: **mCPR would ideally not be the primary indicator for TCI to use to measure progress. mCPR is a slow-changing indicator ill-equipped to capture short-term changes in**

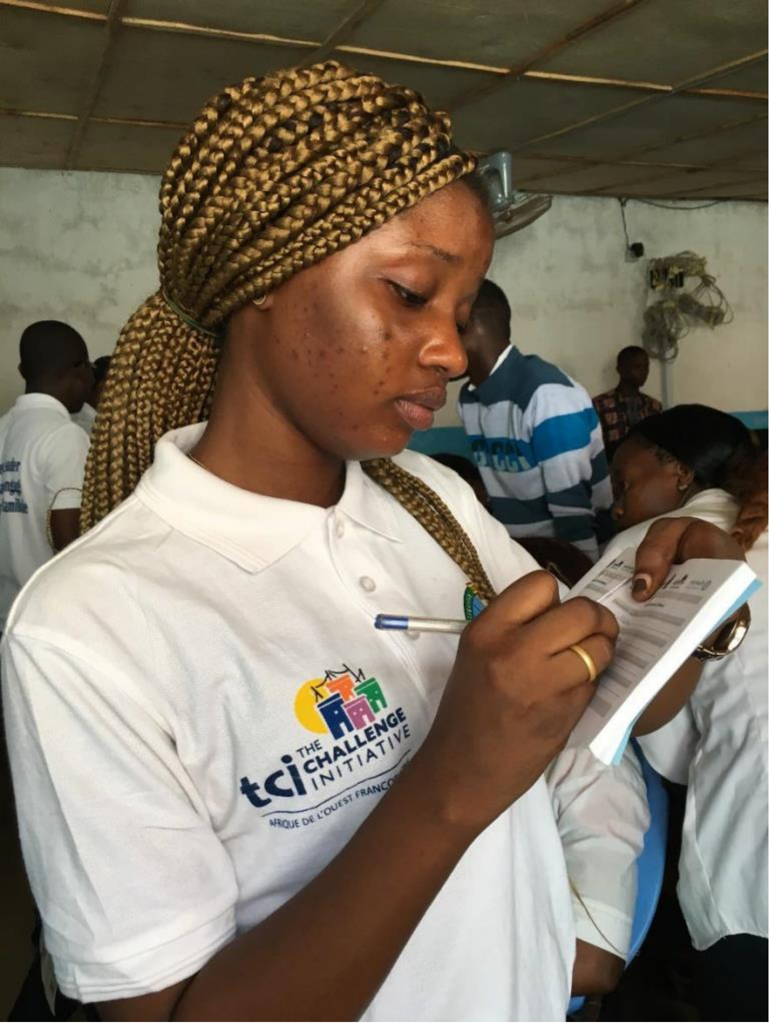

contraceptive coverage. Moreover, since TCI focuses on capacity-building and system strengthening rather than direct implementation, impacts on mCPR may be slow to appear. TCI did seek to leverage PMA Agile data and conduct Local Tracking Surveys and Outcome Tracking Surveys to measure program outputs and intermediate outcomes that might help contextualize trends in mCPR, but our review of these data finds they lack the coverage and consistency needed to track outcomes over time or at the level of TCI geographies.

Impact Findings

Overall, TCI led to limited improvements in contraceptive coverage over the time frame available for the program review, although there was some variation across countries. We were able to estimate impacts on EMU using the synthetic control approach in 16 TCI geographies, out of which 11 had impacts of less than 2 percentage points (Annex 1).[5] This impact roughly translates to less than a 1 percentage point change in contraceptive prevalence at the population level, based on observed trends in EMU and survey-based measures of mCPR across several countries, including the TCI countries in our sample. Descriptive analyses of trends in EMU in the remaining 26 of 42 administrative units (43 of 59 TCI geographies), as well as impact estimates for 7 of these geographies where we have a synthetic control for CYPs per 1,000 WRA, show similarly limited improvements and suggest that TCI likely did not have large impacts on contraceptive coverage during the short time frame (between 18 and 28 months) over which we measured impacts. However, we see some variation across countries, with moderate to large impacts, particularly in Nigeria and Uganda.

All three states in Northern Nigeria (Niger, Kano, and Bauchi) for which we were able to estimate impacts experienced positive impacts on contraceptive coverage[6], although these results need to be interpreted in the context of HMIS data quality issues. However, we found no evidence that TCI had an effect on trends in EMU in Anambra, the one Southern Nigeria state that met our program maturity and data quality criteria to be included in the analysis. Similarly, in Uganda, TCI had large statistically significant impacts on contraceptive coverage in 3 out of 6 districts (Iganga, Mukono, and Buikwe[7]), particularly among youth.[8] However, trend analyses in Kampala and Wakiso suggest that post-TCI trends in EMU remained similar to pre-TCI trends and trends in comparable geographies. In Kenya, there was no evidence of widespread positive impacts of TCI, though there was suggestive evidence of impacts (not statistically significant) in 1 of 5 counties (Uasin Gishu). Similarly, in India, there was little evidence of substantive, positive impacts of TCI. Of the 13 out of 27 districts for which we were able to construct a credible synthetic control for EMU and/or CYPs per 1,000 MWRA in Madhya Pradesh and Uttar Pradesh, only one district (Rewa, Madhya Pradesh) experienced a substantively large, statistically significant impact on EMU, and one district (Gorakhpur, Uttar Pradesh) showed a moderate impact (not statistically significant) on EMU excluding IUCDs.[9]

---

[5] Four TCI geographies had smaller changes in EMU than their synthetic controls, leading to negative impact estimates, but none of these were statistically significant.

[6] We were unable to assess statistical significance in Nigeria given data quality issues limited the number of states in the pool of potential control geographies.

[7] For Buikwe, we found impacts on CYPs per 1,000 WRA.

[8] Because of quality issues affecting data on implants in Uganda, our analysis of EMU excluded implants. However, a separate analysis of implant acceptors in Uganda and supplementary analyses of EMU including implants found that the overall impacts and direction of changes in EMU after the introduction of TCI was not affected by the inclusion of implants.

[9] Because of quality issues affecting data on IUCDs in Uttar Pradesh, our analysis of EMU in Uttar Pradesh excluded IUCDs. The Expert Advisory Group for the Ministry of Health and Family Welfare, with support from Track20, has been exploring the IUCD data quality to improve the ability to interpret trends from HMIS data. However, we conducted a separate analysis of IUCD acceptors in Uttar Pradesh, which found limited impacts. Our analysis on EMU in Madhya Pradesh includes implants.

Due to long-standing donor activity in both TCI and non-TCI geographies, these impact estimates and trend comparisons are most accurately interpreted as the value-added of TCI"s "business unusual" approach relative to the "business as usual" FP programming landscape in each country, rather than the impact of TCI in the absence of any other programming.

Variation in post-TCI growth in and impacts on EMU across countries might be partially explained by pre-TCI contraceptive prevalence, as well as the extent to which TCI addressed geography-specific needs through its programming. Historical trends show that contraceptive use grows in an S-shaped pattern, with geographies with very low or high contraceptive prevalence less likely to have high growth relative to those that have a more moderate prevalence (FP2020). Based on this historical evidence, we would not expect to see similar ranges of growth in EMU across countries, even if TCI were implemented with similar intensity in each geography. Figure 2 depicts the relationship between pre-TCI prevalence for the year in which TCI began in each country, the average post-TCI change in EMU, and the impact estimate for EMU—that is, how much more the EMU grew in the TCI geography relative to its synthetic control, where available.

Overall, post-TCI growth in EMU broadly followed expectations for growth based on a country's position on the S curve, but TCI led to additional growth in some countries. In the three states in Northern Nigeria, which had very low pre-TCI contraceptive prevalence, TCI accelerated growth in EMU relative to their synthetic controls and expectations of limited growth based on historical trends. This finding suggests that TCI's HIIs that alter community norms regarding FP (for example, supporting social mobilization activities and engaging religious leaders) and improve the availability of services (for example, through whole site orientations) were successful in moving the needle on contraceptive growth in Nigeria. In Uganda, although we expected growth in EMU based on the geographies' moderate pre-TCI contraceptive prevalence, we found evidence that TCI may have facilitated higher than anticipated growth. This finding suggests that TCI's HIIs, such as improving contraceptive access through whole site orientations, task shifting, and training of community health workers, supported and accelerated growth in Uganda. Moreover, TCI's AYSRH interventions, which also supported broad aspects of FP, may have been a particularly important component of the Uganda hub's success, given the program's impacts on the youth subpopulation.

On the other hand, in Kenya, post-TCI growth in EMU followed expectations given high pre-TCI contraceptive prevalence and low potential for additional growth, Although TCI counties in Kenya experienced large post-TCI growth in EMU, it was related to recovery of FP service

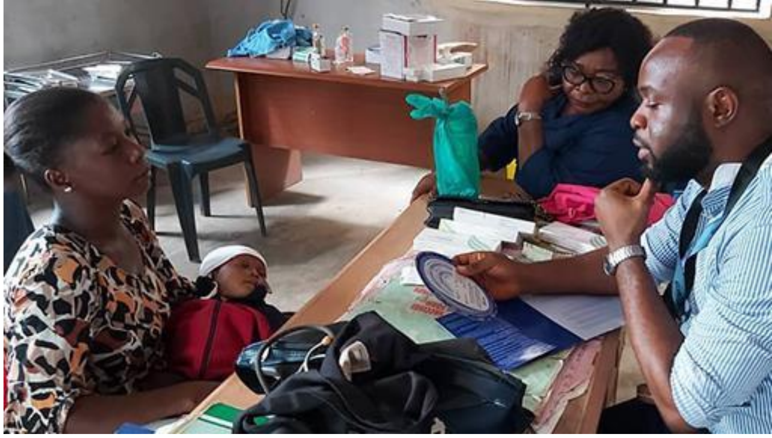

provision following a steep decline during a nationwide strike of doctors and nurses in public facilities that coincided with the beginning of TCI implementation in Kenya. This recovery was observed throughout the country, and we did not detect differential growth in TCI counties. The limited scope for growth in mCPR, as well as ubiquity of FP programming supported by development partners and local policies in most counties in Kenya likely made it challenging for TCI to introduce programming that produced differential results. Finally, in India TCI districts in Madhya Pradesh and Uttar Pradesh showed a wide range of pre-TCI contraceptive prevalence and experienced low to modest post-TCI growth in EMU and limited impacts from TCI. In Madhya Pradesh, these findings are consistent with the high levels of contraceptive prevalence, predominance of birth limiting methods like sterilization, and low median age of sterilization (NFHS4), which limit the scope for additional growth. In Uttar Pradesh, low acceptance of contraceptive methods may have limited the scope for contraceptive growth. Moreover, deep contextual barriers to implementation of HIIs, including staff turnover and shortages in frontline health workers such as ASHAs, particularly in urban areas, as well as facility-level service providers, may have limited differential impacts from TCI.

Trend analyses in nine TCI geographies covered by the PMA Agile data found evidence of positive trends for several key indicators of quality of care in public facilities in India, Kenya, and Nigeria. In Kenya and Nigeria, there were improvements over time in the number of health workers trained in FP, which may reflect the large-scale rollout of TCI's whole site orientations interventions in most TCI facilities. Health facility clients also increasingly reported that they had heard FP messaging from the health facilities they visited, especially in Uttar Pradesh. Consistent with TCI's efforts training health personnel around contraceptive logistics and/or conducting advocacy to boost the stock of commodities at facilities (in Nigeria, East Africa, and India), we saw significant improvements in trends in commodity in-stock status in India and more moderate improvements in two out of three geographies in Kenya. However, Nigeria did not show significant improvements in in-stock status. On the other hand, despite a variety of interventions across TCI hubs that aimed to increase demand for FP services, indicators in related domains showed mixed results, at best.

FIGURE 2. INITIAL PREVALANCE, AVERAGE POST-TCI CHANGE IN EMU, AND IMPACT ESTIMATES

ABOUT THIS FIGURE: The figure includes all geographies that met the criteria for program maturity and data quality. Each point reflects the relationship between the **modeled pre-TCI mCPR on the horizontal axis** and the average **annual post-TCI change in EMU on the vertical axis**. We calculated the annual change by subtracting the average EMU in the 12 months before TCI from the average EMU in 2019 and dividing by the time elapsed between the midpoints of these two 12-month periods.
Circles are color coded according to the legend below. The **size of the circles reflects the estimated impacts of TCI on EMU**, where available. **Circles with a light gray center** indicate the geographies where **no impact estimate** is available; the **outline of the circle reflects to which country or Indian state** the geography belongs. **Circles with red outlines and red text** indicate that the **impact for that geography is negative**; the size of the circle reflects the magnitude of that negative impact.

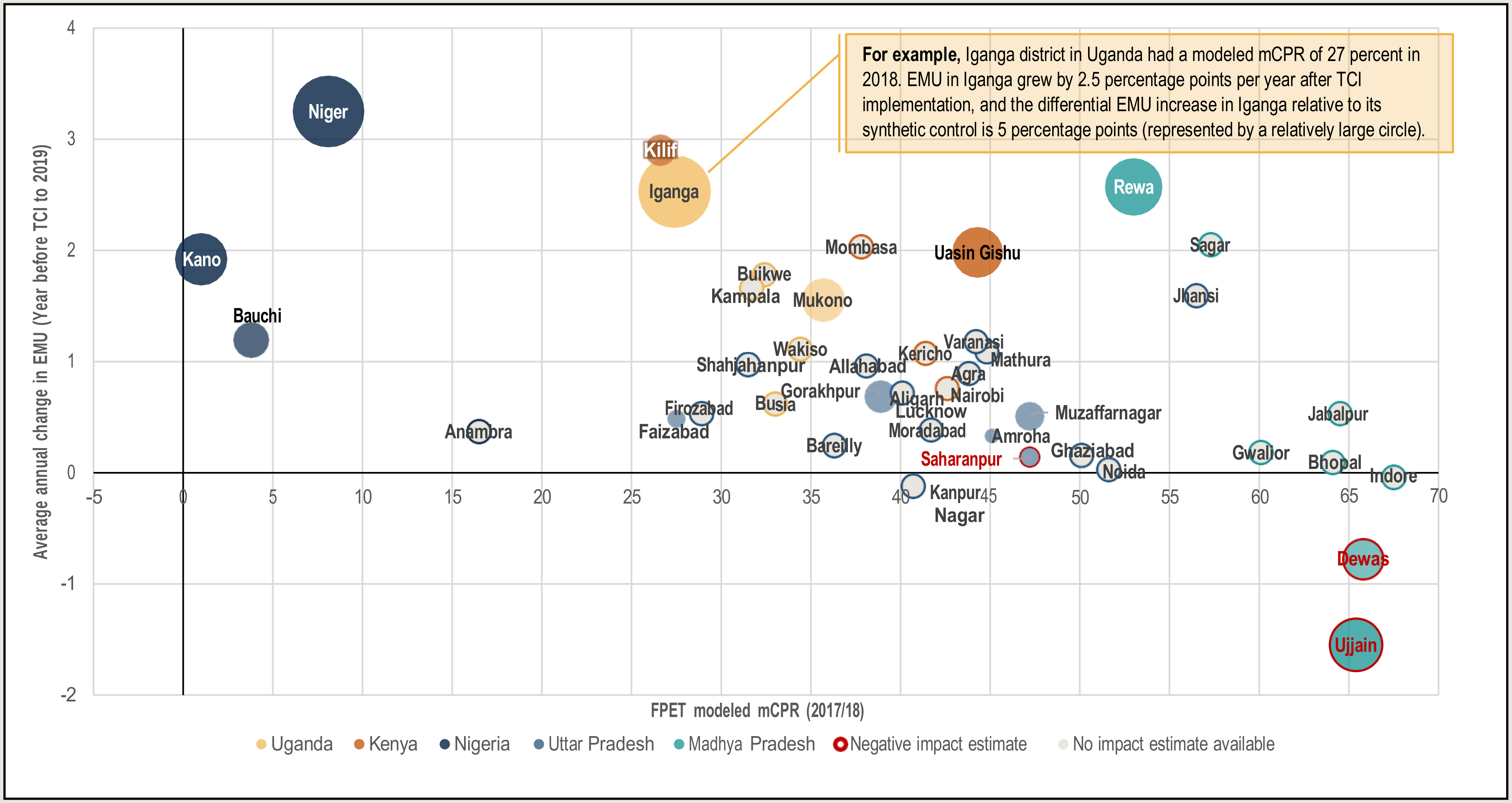

Cost Findings

While some data regarding TCI's cost drivers were available, not all dimensions of the program were tracked. Expenditures by cost category were available for the Gates Institute and the hubs, through their annual financial reports. However, expenditures were not tracked along other dimensions linked to TCI's theory of change, including by activity (for example, development of the toolkits, coaching, facilitation of the community of practice, advocacy, investments in data for decision making, and so on), the HIIs promoted by TCI, or program phase (start-up, implementation, design), although our interviews yielded estimates regarding program phase.

Local governments exceeded those received from the Challenge Fund, and represented 17% of overall funding, but fell short of their overall commitments in some geographies. Contributions by local governments varied, with Nigeria and India governments contributing the highest share relative to their commitments and local governments in FWA and East Africa fulfilling about half of their commitments over years 2-4. This has implications for sustainability, as the objective of TCI is to move programs to full local government funding within approximately two years.

TCI's capacity-building approach to sustainable institutionalization of service delivery improvements is relatively resource intensive, but in a few geographies the program appeared to be relatively cost-efficient. Due to the diversity of population sizes served in each geography—ranging from Indian cities and Nigerian states with several million women of reproductive age to municipal jurisdictions and counties with less than 100,000 women of reproductive age in East Africa—it is helpful to look at standardized metrics such as cost per additional user of modern contraceptives or cost per CYP. As shown in Figure 3, several TCI geographies appear to be within the range of other FP program approaches on the basis of cost per CYP at less than $20 per CYP in Uasin Gishu, Gorakhpur, Rewa and Kano (though among these, only Rewa is based on differences in impacts that were statistically significant). Further, our econometric analyses suggest that TCI experienced economies of scale at higher levels of output although we did not find evidence of learning-by-doing over the first four years of TCI implementation.

FIGURE 3. COST PER CYP

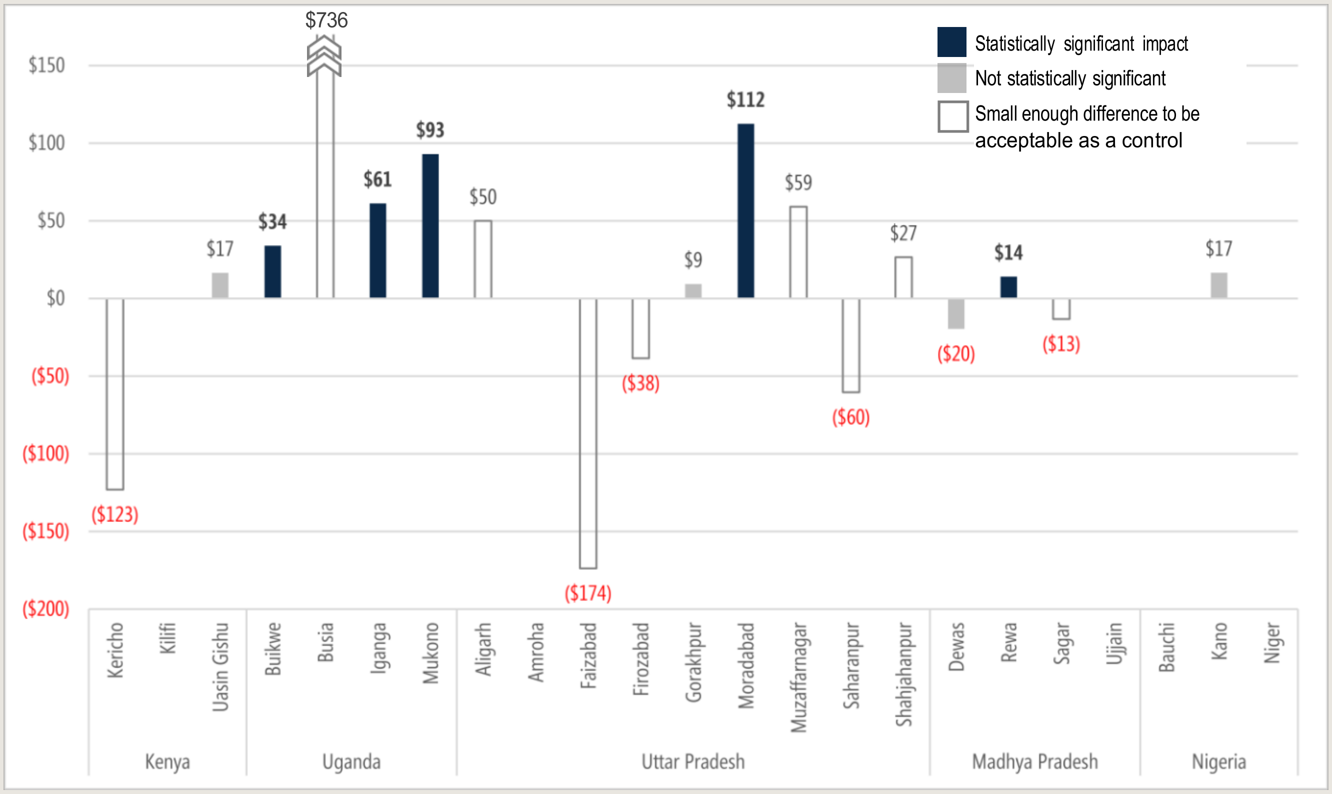

Reflections

TCI's commitment to systems strengthening and capacity building may be somewhat in conflict with the potential for achieving impacts quickly on FP outcomes. Most geographies had been implementing for 18 months to two years as of year 4 of the project, but this time period was used not only for on-the-ground implementation of supply- and demand-side HIIs, but also ongoing coaching and systems strengthening efforts. Because increased contraceptive uptake depends in part on improving intermediate outcomes, this range is a relatively short time horizon for assessing the impacts of a program like TCI on contraceptive prevalence. Identifying and measuring intermediate outcomes towards the pathway to change will provide a signal of whether progress is being made.

Consider differentiating how "success" is defined across implementation contexts. In geographies with high baseline contraceptive prevalence, targeting other outcomes may be more appropriate, e.g. equity in service provision, or expanding method choice.

The number and diversity of the combinations of HIIs being implemented across 94 geographies in 10 countries make it challenging to generate learning across the TCI portfolio on what may be driving program impacts and costs. The ability for regions and geographies to fit best practices to the gaps they identified in their EOIs and project designs was a great part of the appeal of TCI. However, this diversity of programs, combined with the program monitoring data approaches that were inconsistent across hubs, makes it more difficult to learn about what worked and in what context for specific HIIs, as well as what they cost to implement.

References: United Nations Department of Economic and Social Affairs. "68% of the world population projected to live in urban areas by 2050, says UN." May 16, 2018. Accessed: March 31, 2021.

The TCI program review was led by Mathematica and Avenir Health. Authors are listed below. Photos are courtesy of TCI.

Project Lead	Impact team		Implementation/Sustainability team	Cost-effectiveness team
Nancy Murray	Anitha Sivasankaran<br>Laura Meyer<br>Alejandra Aponte<br>Evan Borkum	Emily Sonneveldt<br>Priya Emmart<br>Rebecca Rosenberg	Swetha Sridharan<br>Beryl Seiler<br>Sara Bryk<br>Lisa DeMaria<br>Sydney Taylor	Lori Bollinger<br>Kristin Bietsch

For the full report, please visit www.mathematica.org.

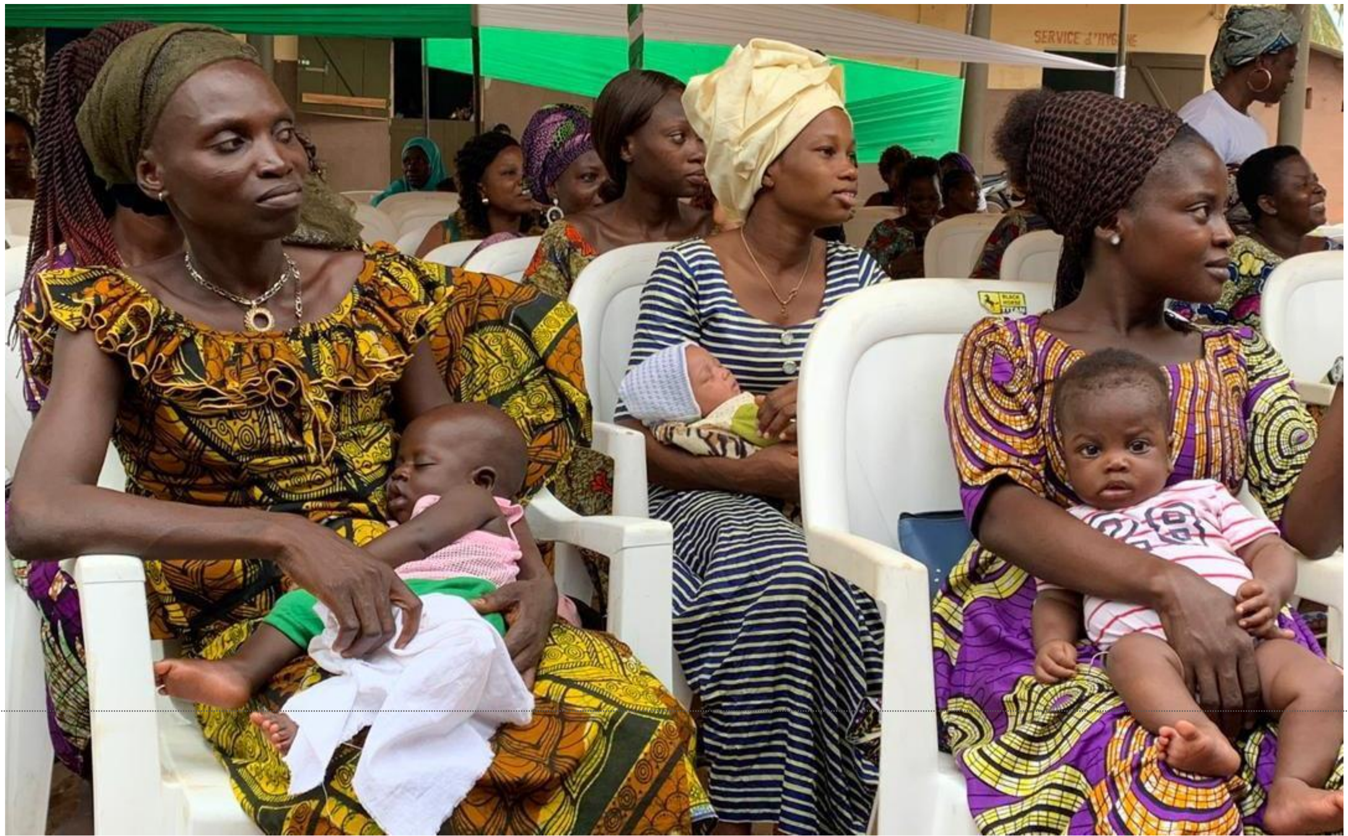

ANNEX. TRENDS IN EMU, WITH SYNTHETIC CONTROLS, WHERE AVAILABLE

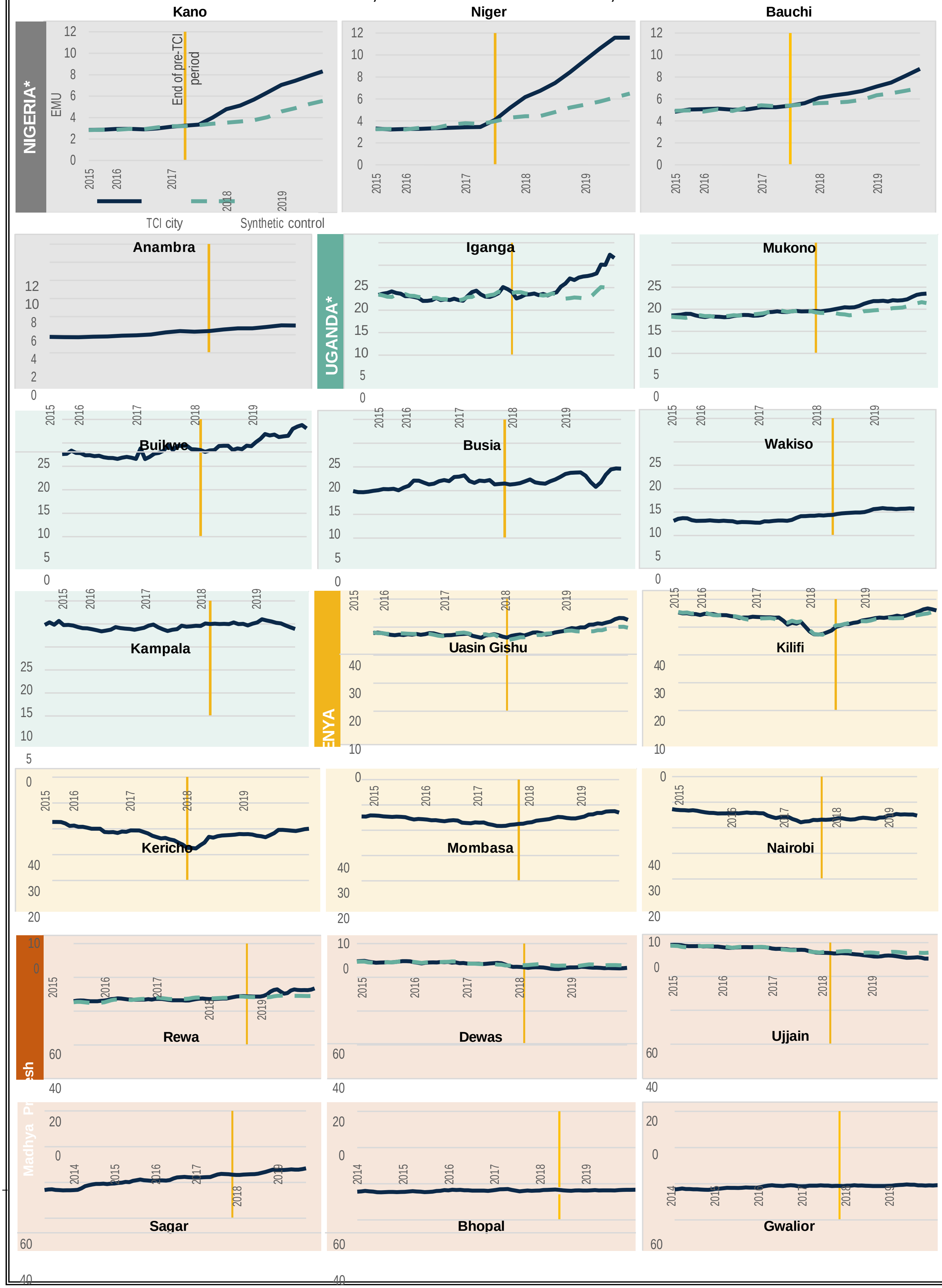

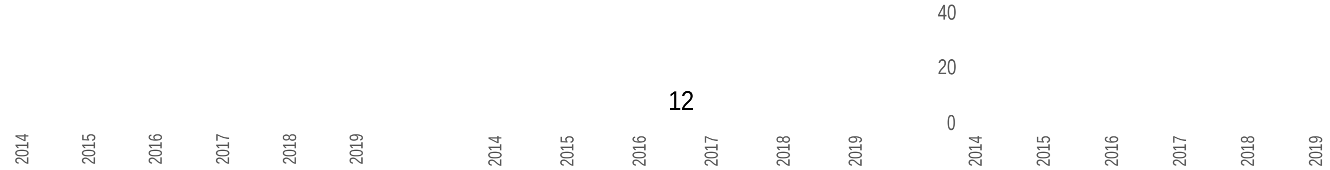

* Our analyses for Nigeria and Uganda start in July 2015 due to gaps in data quality and availability. EMU excludes implants in Uganda.

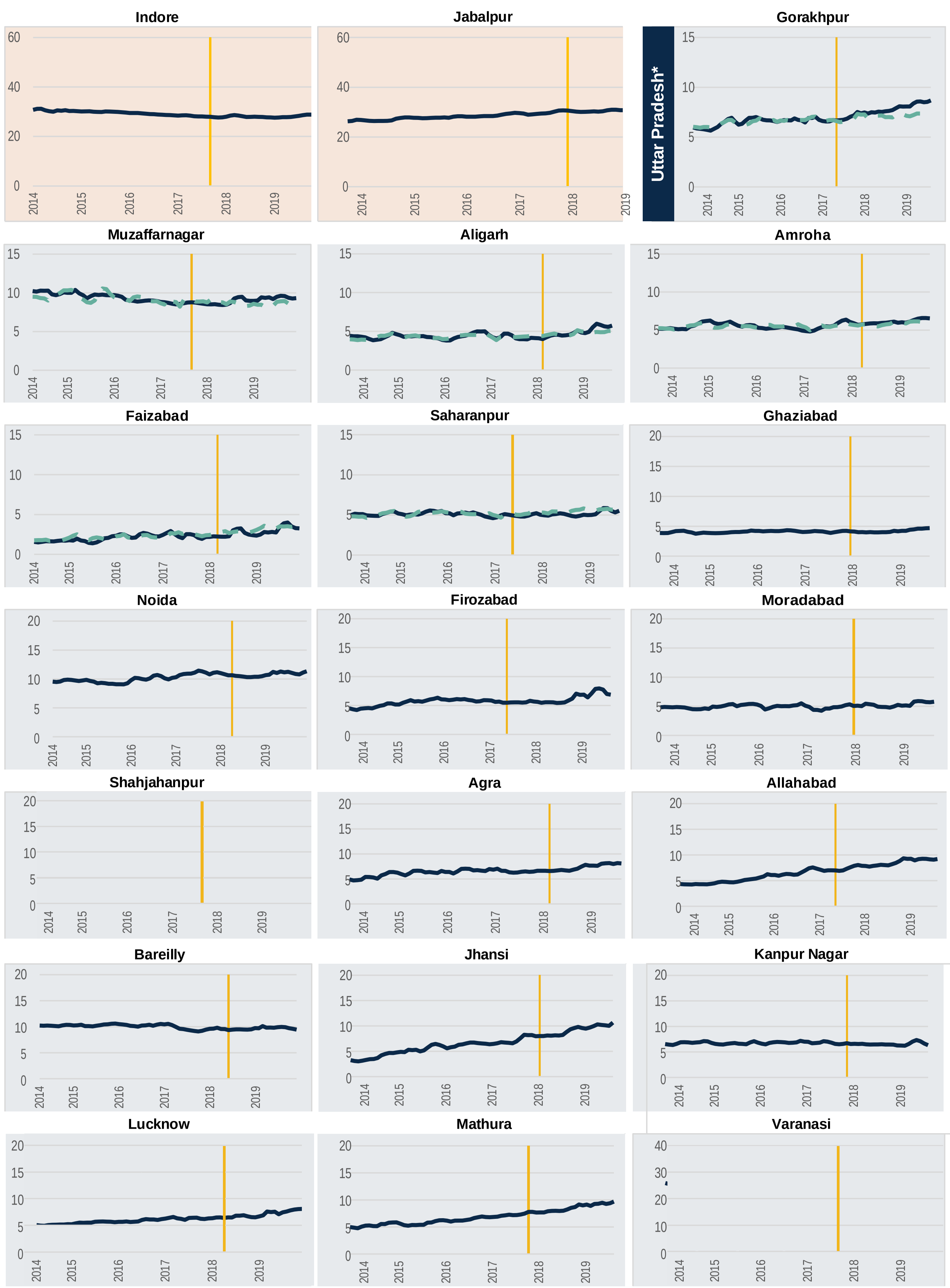

* Our analyses for Uttar Pradesh start in April 2014 due to gaps in data quality and availability. EMU excludes IUCD in UP.

Supplement to: Finkle C, Martin K, Salas I, et al. A platform for sustainable scale: The Challenge Initiative's innovative approach to scaling proven interventions. *Glob Health Sci Pract*. 2023;11(Suppl 1):e2200167. https://doi.org/10.9745/GHSP-D-22-00167

Supplement 4

TCI's Refreshed Key Performance Indicators (KPIs)

1. Number of local governments implementing TCI FP programs, including population footprint
2. Amount of annual funds committed and budgeted by local governments to implement TCI programs
3. Number of FP and AYSRH primary interventions conducted by the local government with support from TCI
4. Number/Percentage of local governments with a well-functioning coaching program on FP and AYSRH interventions
5. Number of FP clients, by method type (short-acting, long-acting reversible, or permanent methods)
6. Percentage of women ages 15–49 years who currently use a modern contraceptive method (where surveys are available)
7. Percentage of women ages 15–49 years who intend to use a modern contraceptive method in next 12 months (where surveys are available)
8. Number of local governments that graduate from TCI, i.e. transitioned to coaching on demand
9. Amount of funds raised from donors for TCI programs
10. Number of actionable recommendations developed to inform decisions or to take corrective action as a result of learning and reflection exercises, by Hub and Global teams
11. Number of publications produced by Hub and Global author teams to contribute to the field of sustainable scaling in global health and development
